# Diprenylated cyclodipeptide production by changing the prenylation sequence of the nature’s synthetic machinery

**DOI:** 10.1007/s00253-022-12303-4

**Published:** 2022-11-28

**Authors:** Wen Li, Lindsay Coby, Jing Zhou, Shu-Ming Li

**Affiliations:** Institut Für Pharmazeutische Biologie Und Biotechnologie, Fachbereich Pharmazie, Philipps-Universität Marburg, Robert-Koch Straße 4, 35037 Marburg, Germany

**Keywords:** Cyclodipeptides, Multiple prenylation, Indole prenyltransferases, EchPT1, Chemoenzymatic synthesis

## Abstract

**Abstract:**

Ascomycetous fungi are often found in agricultural products and foods as contaminants. They produce hazardous mycotoxins for human and animals. On the other hand, the fungal metabolites including mycotoxins are important drug candidates and the enzymes involved in the biosynthesis of these compounds are valuable biocatalysts for production of designed compounds. One of the enzyme groups are members of the dimethylallyl tryptophan synthase superfamily, which mainly catalyze prenylations of tryptophan and tryptophan-containing cyclodipeptides (CDPs). Decoration of CDPs in the biosynthesis of multiple prenylated metabolites in nature is usually initiated by regiospecific *C2*-prenylation at the indole ring, followed by second and third ones as well as by other modifications. However, the strict substrate specificity can prohibit the further prenylation of unnatural *C2*-prenylated compounds. To overcome this, we firstly obtained *C4*-, *C5*-, *C6*-, and *C7*-prenylated *cyclo*-l-Trp-l-Pro. These products were then used as substrates for the promiscuous *C2*-prenyltransferase EchPT1, which normally uses the unprenylated CDPs as substrates. Four unnatural diprenylated *cyclo*-l-Trp-l-Pro including the unique unexpected *N1*,*C6*-diprenylated derivative with significant yields were obtained in this way. Our study provides an excellent example for increasing structural diversity by reprogramming the reaction orders of natural biosynthetic pathways. Furthermore, this is the first report that EchPT1 can also catalyze *N1*-prenylation at the indole ring.

**Key points:**

*• Prenyltransferases as biocatalysts for unnatural substrates.*

*• Chemoenzymatic synthesis of designed molecules.*

*• A cyclodipeptide prenyltransferase as prenylating enzyme of already prenylated products.*

**Graphical Abstract:**

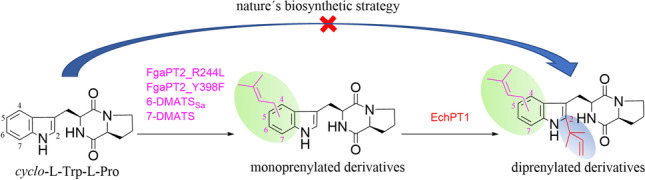

**Supplementary Information:**

The online version contains supplementary material available at 10.1007/s00253-022-12303-4.

## Introduction

Microorganisms, especially ascomycetous fungi, are often found in contaminated agricultural products like food crops including corns, grain, fruits, and vegetables as well as poorly conserved foods. They produce diverse mycotoxins which are hazardous to human and animal health (Dey et al. 2022; Nan et al. 2022; Pallares et al. 2022). On the other hand, the microbial natural products including mycotoxins are important drug candidates (Newman 2021) and the enzymes involved in the biosynthesis of these compounds are valuable biocatalysts for structural modification and construction of new biosynthetic pathways (Yi et al. 2021). Among them, tailoring enzymes for modification of the backbone skeletons play key roles in increasing structural diversity and biological activities (Harken and Li 2021; Yang et al. 2022). Prenyltransferases belong to one of the important modification enzyme groups and catalyze transfer of nxC_5_ moieties onto different accepters (Winkelblech et al. 2015). Members of the dimethylallyl tryptophan synthase (DMATS) superfamily are most investigated prenyltransferases in the last years. They mainly catalyze prenylations of tryptophan and tryptophan-containing cyclodipeptides (CDPs) by using dimethylallyl diphosphate (DMAPP) as prenyl donor and are involved in the biosynthesis of a large number of prenylated indole alkaloids including mycotoxins (Winkelblech et al. 2015).

**Fig. 1:**
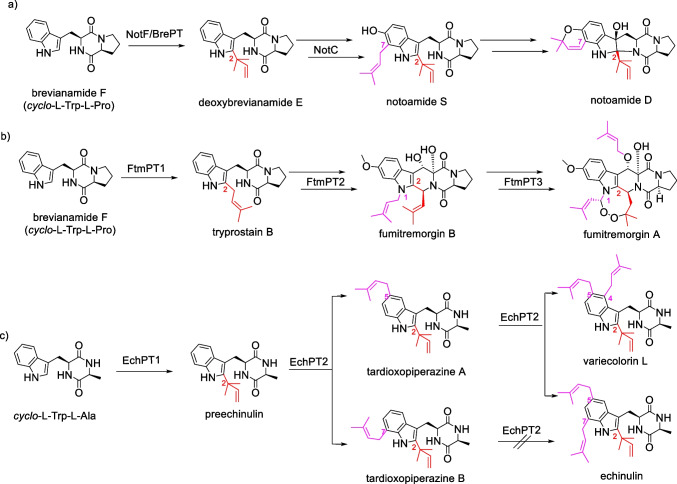
Examples of biosynthetic pathways for multiprenylated CDP derivatives with *C2*-prenylation as the first decoration step

Prenylated indole alkaloids are widespread in bacteria, fungi, plants, and marine organisms (Klas et al. 2018; Li 2010; Yazaki et al. 2009) and exhibit clearly distinct biological activities from their nonprenylated precursors (Botta et al. 2005; Wollinsky et al. 2012). Prenylated tryptophan-containing cyclodipeptides and derivatives thereof represent an important category within the prenylated indole alkaloids. As examplified in Fig. 1, the cytotoxic notoamides from *Penicillium* and *Aspergillus* species are derivatives of *C2*,*C7*-diprenylated and *C6*-hydroxylated brevianamide F (*cyclo*-l-Trp-l-Pro) (Klas et al. 2018). Fumitremorgins as di- and triprenylated brevianamide F products were identified as tremorgenic metabolites in *Aspergillus fumigatus*, *Neosartorya fischeri*, and other fungi (Li 2010; Mundt et al. 2012). Di- and triprenylated *cyclo*-l-Trp-l-Ala and congeners also occur frequently in the fungal genera of *Aspergillus* and *Eurotium* with echinulin as the important representative (Chen et al. 2015; Du et al. 2017; Kamauchi et al. 2016; Li 2010; Nies and Li 2021; Wohlgemuth et al. 2017).

Biosynthetically, the skeletons of such CDPs in fungi are usually assembled by nonribosomal peptide synthases using tryptophan and a second amino acid as substrates. The CDP core is then decorated by different tailoring enzymes including prenyltransferases from the DMATS superfamily (Li 2010; Wohlgemuth et al. 2017; Xu et al. 2014). In nature, CDPs are mostly monoprenylated at positions C-2 and C-3, and occasionally at positions C-4 to C-7. In contrast, prenylation of the free amino acid tryptophan takes place more frequently at C-4 to C-7 of the benzene ring than other positions (Winkelblech et al. 2015). As shown in Fig. 1, the biosynthesis of multiple prenylated CDPs is usually initiated by the regiospecific *C2*-prenylation at the indole ring, followed directly by the second prenyltransferase as in the case of echinulins or after decoration with other enzymes, e.g., in the biosynthesis of notoamides and fumitremorgins (Klas et al. 2018; Li 2011; Wohlgemuth et al. 2017). Remarkably, the members of the DMATS superfamily show high substrate flexibility toward their aromatic substrates and accept not only structurally similar, but also distinct compounds as prenyl acceptors. For example, the brevianamide F *C2*-prenyltransferases FtmPT1 and BrePT as well as the *cyclo*-l-Trp-l-Ala *C2*-prenyltransferase EchPT1 (Fig. 1) accept well other CDPs for *C2*-prenylation (Grundmann and Li 2005; Wohlgemuth et al. 2017; Wollinsky et al. 2012; Yin et al. 2013). EchPT2 from the echinulin biosynthetic pathway (Fig. 1) converts *C2*-prenylated *cyclo*-Trp-Ala and *cyclo*-Trp-Pro isomers to tri- and tetraprenylated derivatives (Wohlgemuth et al. 2018). Diprenylated products, e.g., tardioxopiperazines A and B, were only detected as minor products. Furthermore, the tryptophan *C6*-prenyltransferase from *Streptomyces ambofaciens* and 7-DMATS from *Aspergillus fumigatus* can also prenylate CDPs at positions C-6 and C-7, respectively (Kremer et al. 2007; Liu et al. 2020; Winkelblech and Li 2014). In addition, protein engineering of the tryptophan *C4*-prenyltransferase from *Aspergillus fumigatus* led to the mutant FgaPT2_R244L with increased acceptance for CDPs (Fan and Li 2016). As a proof of concept, we decided in this study to produce unnatural *C2,C4*-, *C2,C5*-, *C2,C6*-, and *C2,C7*-diprenylated *cyclo*-l-Trp-l-Pro by combination of different prenyltransferases.

## Materials and methods

### Chemicals

DMAPP was chemically prepared according to the method published previously (Woodside et al. 1988). *cyclo*-l-Trp-l-Pro was synthesized as described in literature (Caballero et al. 1998).

### Bacterial strains, plasmids, and culture conditions

*Escherichia coli* strains M15 [pREP4] (Qiagen, Hilden, Germany) and BL21 (DE3) pLysS (Invitrogen, Karlsruhe, Germany) harboring the plasmids pVW90, pALF49, pPM37, pJW12, and pLW40 were used for overproduction of the recombinant proteins EchPT1, FgaPT2_R244L, FgaPT2_Y398F, 6-DMATS_Sa_, and 7-DMATS, respectively (Fan and Li 2016; Kremer et al. 2007; Mai et al. 2016; Winkelblech and Li 2014; Wohlgemuth et al. 2017). Terrific broth (TB) medium supplemented with 50 µg/mL carbenicillin or 25 µg/mL kanamycin was used for cultivation of recombinant *E. coli* strains.

### Protein overproduction and purification as well as enzyme assays

**Fig. 2:**
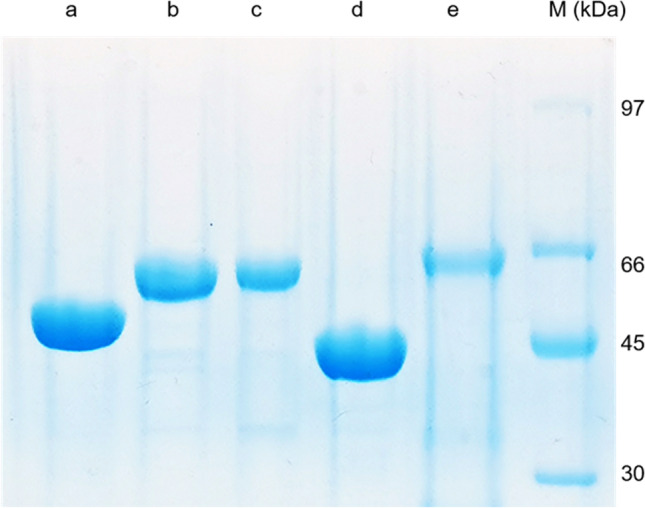
SDS-PAGE analysis of the purified prenyltransferases. The proteins were separated on a 12% polyacrylamide gel and stained with Coomassie brilliant blue R-250. Lanes from left to right: a, EchPT1; b, FgaPT2_R244L; c, FgaPT2_Y398F; d, 6-DMATS_Sa_; e, 7-DMATS; M, protein marker

Culture conditions and purification of His_6_-EchPT1, His_8_-FgaPT2_R244L, His_8_-FgaPT2_Y398F, 6-DMATS_Sa_-His_6_, and 7-DMATS-His_6_ were carried out by Ni–NTA affinity chromatography (Qiagen, Hilden) as described previously (Fan and Li 2016; Kremer et al. 2007; Mai et al. 2016; Winkelblech and Li 2014; Wohlgemuth et al. 2017). SDS-PAGE analysis revealed that all the five recombinant proteins were purified to near homogeneity (Fig. 2).

To determine the enzyme activity, standard assays (50 µL) contained Tris–HCl (50 mM, pH 7.5), aromatic substrate (1 mM), DMAPP (1 mM), glycerol (0.5–5%, v/v), DMSO (2.5%, v/v), and the purified protein (4 µg). CaCl_2_ at a final concentration of 5 mM was added to the reaction mixtures to enhance the reaction velocity (Li 2009; Sasaki et al. 2008). After incubation at 37 °C for 16 h, the reaction mixtures were terminated by addition of 50 µL methanol. The precipitated proteins were removed by centrifugation at 13,000 × g for 20 min and analyzed on liquid chromatography coupled with mass spectrometer (LCMS) as described below.

The linearity of the EchPT1 reactions toward monoprenylated derivatives was determined up to 240 min with 4 µg protein. The assays for determination of the kinetic parameters of EchPT1 toward monoprenylated CDPs **3**, **4**, and **5** contained 1 mM DMAPP, 4 µg EchPT1 and the aromatic substrate at final concentrations of 0.01, 0.02, 0.05, 0.1, 0.2, 0.5, 1.0, and 2.0 mM. The reaction mixtures containing **3**, **4**, and **5** were incubated at 37 °C for 30, 35, and 30 min, respectively. For kinetic parameters toward DMAPP of EchPT1 in the presence of the monoprenylated compound **3**, the reaction mixture contained 4 µg EchPT1, **3** (1 mM), CaCl_2_ (5 mM), and DMAPP at final concentrations from 0.01 to 2.0 mM, which was incubated at 37 °C for 30 min. All the assays were performed as triplicates and subsequently terminated with methanol, and analyzed on high performance liquid chromatography (HPLC) as described below. The conversion yields were calculated by using the isolated products as standards or by ratio of the peak areas of product and substrate in HPLC chromatograms. The data were fitted to the Michaelis–Menten equation in Prism 4.0 (GraphPad Software).

### Preparation and isolation of the enzyme products for structure elucidation

Enzyme assays for product isolation were scaled up to a volume of 12 mL, containing Tris–HCl (50 mM, pH 7.5), DMAPP (1.5 mM), the respective aromatic substrate (1 mM), and 1–5 mg purified recombinant proteins. The reaction mixtures were incubated at 37 °C for 16 h and extracted three times with two volumes of ethyl acetate each. The resulting organic phases were combined and concentrated on a rotating vacuum evaporator at 35 °C to dryness and dissolved in 1 ml methanol for isolation.

### LCMS and HPLC conditions for analysis and isolation of the enzyme products

LCMS analysis was performed as described previously (Zhou and Li 2021). The substances were eluted at a flow rate of 0.25 mL/min with a linear gradient from 5 to 100% ACN in 10 min. Semi-preparative HPLC was performed with an Agilent Eclipse XDB-C18 (250 × 9.4 mm, 5 µm) column. Water (A) and acetonitrile (B) were used as solvents at flow rate of 2 mL/min. Compounds **2**, **3**, **4**, **5**, and **6** were isolated with 55% B, **10** with 60% B and **7**, **8**, and **9** with 65% B.

### Nuclear magnetic resonance analysis

NMR spectra were recorded on a JEOL ECA-500 MHz spectrometer (JEOL, Tokyo, Japan) and processed with MestReNova 6.1.0 (Metrelab). Chemical shifts were referred to those of the solvent signals. The NMR data are provided in Tables S1–S4 and spectra in Figs. S1–S25.

## Results

### Attempts to produce diprenylated derivatives by prenylation of already *C2*-prenylated cyclo-L-Trp-L-Pro

**Fig. 3:**
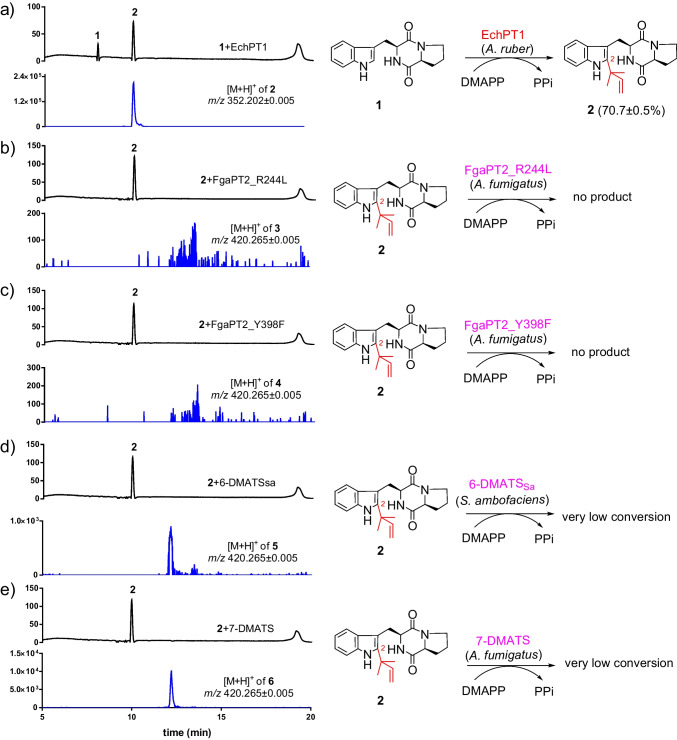
**a** LCMS analysis of the acceptance of *cyclo*-l-Trp-l-Pro (**1**) by EchPT1. **b**–**e** LCMS analysis of the acceptance of deoxybrevianamide E (**2**) by FgaPT2_R244L, FgaPT2_Y398F, 6-DMATS_Sa_, and 7-DMATS. UV absorptions at 280 nm are illustrated. All the assays were performed in duplicates. The conversion yield with EchPT1 is given as the mean value

To get *C2,C4*-, *C2,C5*-, *C2,C6*-, and *C2,C7*-diprenylated *cyclo*-l-Trp-l-Pro, we first followed the logic of the nature’s biosynthetic strategy by using the reverse *C2*-prenyltransferase EchPT1 from *A. ruber* (Wohlgemuth et al. 2017) as the first biocatalyst for prenylation of *cyclo*-l-Trp-l-Pro (**1**). The obtained *C2*-prenylated derivative deoxybrevianamide E (**2**) should be then used as substrate for prenylation at *C4*-, *C5*-, *C6*-, and *C7* of the benzene ring with other prenyltransferases. The results of the enzyme assay are shown in Fig. 3. LCMS analysis revealed that **1** was well converted to **2** having a [M + H]^+^ ion at *m/z* 352.2023 by EchPT1 with a conversion yield of 70.7 ± 0.5% (Fig. 3a). Isolation on HPLC and comparison of its ^1^H NMR data (Fig. S1) with those published previously (Schkeryantz et al. 1999) confirmed **2** to be the expected deoxybrevianamide E.

To obtain the desired diprenylated derivatives, **2** was used as substrate for the second prenylation. As mentioned in the introduction, FgaPT2_R244L, 6-DMATS_Sa_, and 7-DMATS catalyze *C4*-, *C6*-, and *C7*-prenylation of CDPs, respectively (Fan and Li 2016; Kremer et al. 2007; Winkelblech and Li 2014). In a previous study, we have demonstrated that the mutant FgaPT2_Y398F catalyzed the *C4*- and *C5*-penylations at the indole ring of the tripeptide derivative ardeemin FQ (Mai et al. 2016). These four enzymes were chosen for prenylation of **2** at the positions of C-4, C-5, C-6, and C-7 of the benzene ring, respectively. Unfortunately, LCMS analysis revealed that no products were detected in the incubation mixtures of FgaPT2_R244L and FgaPT2_Y398F. Formation of diprenylated products with [M + H]^+^ ions at *m/z* 420.265 ± 0.005 was observed in the assays with 6-DMATS_Sa_ and 7-DMATS, but only detected in the extracted ion chromatograms (EICs) (Fig. 3b–e). Obviously, prenylation at C-2 of **1** prohibited its acceptance by the tested enzymes. Such low yields make almost impossible to get enough products for structural elucidation. Therefore, we changed our strategy and tested the possibility with first prenylation at the benzene ring by FgaPT2_R244L, FgaPT2_Y398F, 6-DMATS_Sa_, and 7-DMATS, followed by the *C2*-prenylation of the monoprenylated products with EchPT1.

### Prenylation of cyclo-L-Trp-L-Pro at the benzene ring by indole prenyltransferases

**Fig. 4:**
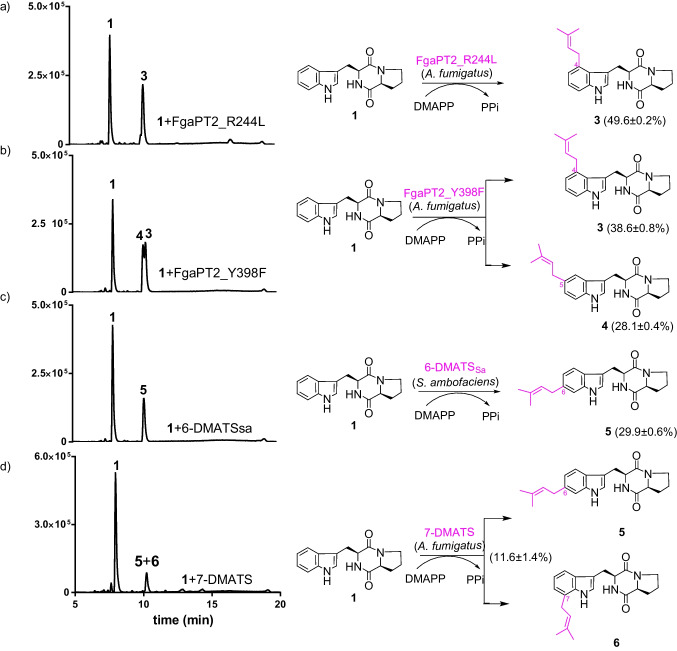
LCMS analysis of *cyclo*-l-Trp-l-Pro (**1**) prenylation by **a** FgaPT2_R244L, **b** FgaPT2_Y398F, **c** 6-DMATS_Sa_, and **d** 7-DMATS. Only absorptions at 280 nm are illustrated. All the assays were performed in duplicates. The conversion yields are given as mean values

To obtain diprenylated derivatives in a different way from nature, substrate **1** was incubated with FgaPT2_R244L, FgaPT2_Y398F, 6-DMATS_Sa_, and 7-DMATS in the presence of DMAPP at 37 °C for 16 h. LCMS analysis of the reaction mixtures revealed the conversion of **1** to monoprenylated products with [M + H]^+^ ions at *m/z* 352.202 ± 0.005 in all the four assays. One product peak each was detected in the reaction mixtures of FgaPT2_R244L, 6-DMATS_Sa_, and 7-DMATS with conversion yields of 49.6 ± 0.2, 29.9 ± 0.6, and 11.6 ± 1.4%, respectively (Fig. 4). Two peaks with a total conversion yield of 66.7 ± 0.8% were observed in the assay with FgaPT2_Y398F. Isolation and structural elucidation proved the product of FgaPT2_R244L to be *C4*-prenylated derivative **3** (see below for structural elucidation). Both *C4*- (**3**) and *C5*-prenylated derivative **4** were isolated and identified from the reaction mixture of FgaPT2_Y398F. The sole product of 6-DMATS_Sa_ was the expected *C6*-prenylated *cyclo*-l-Trp-l-Pro (**5**). NMR analysis of the product peak of the enzyme assay with 7-DMATS proved the presence of both **5** and the *C7*-prenylated product **6** in a ratio of 0.6:1.0. Prenylation by 7-DMATS at positions C-6 and C-7 of the benzene ring was already described for *cyclo*-l-Homotrp-d-Val (Fan and Li 2013). Due to their similar physiochemical properties, **5** and **6** could not be separated from each other. Nevertheless, their structures can be unequivocally elucidated by NMR analysis, because of the availability of the spectrum for **5** in high purity obtained from the enzyme assay with 6-DMATS_Sa_.

### Confirmation of the prenylation positions of the monoprenylated products

For structural elucidation, the enzyme products were isolated on a preparative scale and their structures were elucidated by MS and NMR analyses. ESI–MS proved that the products of **1** with FgaPT2_R244L, FgaPT2_Y398F, 6-DMATS_Sa_, and 7-DMATS had [M + H]^+^ ions at *m/z* 352.202 ± 0.005, 68 Dalton larger than that of **1**, indicating attachment of a dimethylallyl moiety to their structures. This hypothesis was confirmed by appearance of signals for a regular dimethylallyl residue each at δ_H_ 3.4–3.7 (d or dd, 2H-1´), 5.3–5.4 (tsept, H-2´), and 1.7–1.8 (one or two br s, 3H-4´ and 3H-5´) (Figs. S2–S4 and S9). The ^1^H NMR spectra of the isolated compounds showed in the aromatic region signals for four instead of five protons in that of **1**. The signal for H-2 was detected in all of the spectra of the isolated products **3**–**6**, indicating the prenylation at the benzene ring. Three vicinal aromatic protons were found in the spectra of **3** and **6**, suggesting for the *C4*- and *C7*-prenylation. Coupling pattern of the three aromatic protons in **4** and **5**, *i.e.*, one at *meta*- and two at *ortho*-position, is consistent with *C5*- and *C6*-prenylations. The structure of compound **5** was unequivocally determined as *C6*-prenylated *cyclo*-l-Trp-l-Pro by interpretation of its ^1^H, ^13^C, ^1^H-^1^H COSY, HSQC, and HMBC NMR data (Figs. S4–S8). Clear long-range correlations between H-5 and C-9 as well as H-7 and C-9 were observed in the HMBC spectrum, proving the attachment of the prenyl moiety at C-6 of the indole ring (Fig. 4). Comparison of the NMR data of **3** and **6** with those published previously proved their structures to be *C4*- and *C7*-prenylated derivatives, respectively (Fig. 4) (Liu et al. 2020; Steffan and Li 2009).

### Prenylation of the C4-, C5-, C6-, and C7-prenylated cyclo-L-Trp-L-Pro by EchPT1 and structural elucidation of the diprenylated products

**Fig. 5:**
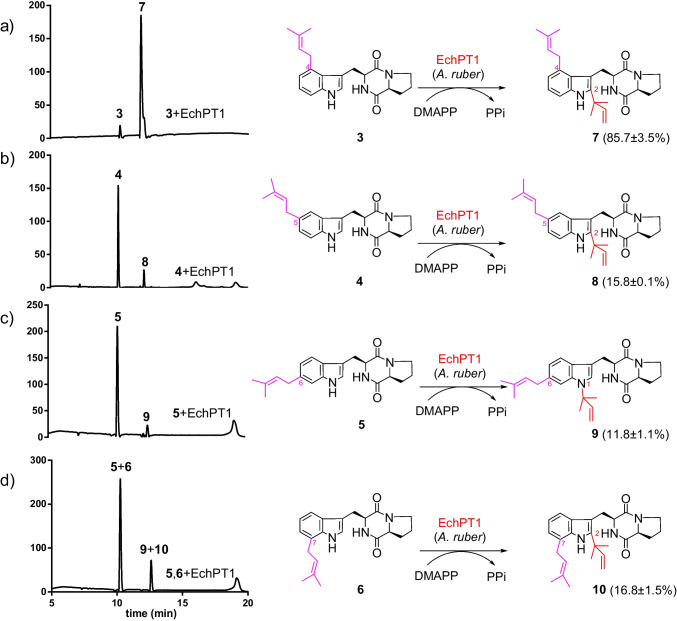
LCMS analysis of acceptance of monoprenylated derivatives (**3**, **4**, **5**, and **6**) by EchPT1. Products **9** and **10** with a total conversion yield of 23.6 ± 1.4% were isolated from the incubation mixture of **6** with **5**. Absorptions at 280 nm are illustrated. All the assays were performed in duplicates. The conversion yields are given as mean values

The isolated monoprenylated samples from the enzyme assays of **1** with FgaPT2_R244L, FgaPT2_Y398F, 6-DMATS_Sa_, and 7-DMATS, *i.e*., **3**, **4**, and **5** in high purity and **6** in a mixture with **5**, were used for incubation with the *cyclo*-l-Trp-l-Ala reverse *C2*-prenyltransferase EchPT1 in the presence of DMAPP. LCMS analysis showed all these substrates were accepted by EchPT1 with the highest conversion of 85.7 ± 3.5% for **3** (Fig. 5). Only one product peak each was detected, even in the assay with the mixture of **5** and **6** (Fig. 5d). Isolation and structural elucidation confirmed the presence of one product each in the incubation mixtures of **3**–**5**. As expected, the single product peak of the incubation mixture of **5** and **6** comprised two products, which can be separated from each other under optimized conditions.

The isolated products **7**–**10** from the enzyme assays with **3**–**6** were then subjected to MS and NMR analyses. ESI–MS proved that all these new products were diprenylated *cyclo*-l-Trp-l-Pro with [M + H]^+^ ions at *m/z* 420.265 ± 0.005. In the ^1^H NMR spectra of **7**, **8**, and **10**, the signal for H-2 disappeared. Instead, additional signals for a reverse dimethylallyl moiety were detected at δ_H_ 6.12–6.14 (dd, 17, 10 Hz, H-2˝), 5.17–5.20 (dd, 17, 0.6 Hz, H-3˝), 5.16–5.23 (dd, 10, 0.6 Hz, H-3˝), and 1.5–1.7 (two singlets, 3H-4˝and 3H-5˝) (Figs. S10, S15, and S24). These data suggested **7**, **8**, and **10** to be *C2,C4*-, *C2,C5*-, and *C2,C7*-diprenylated *cyclo*-l-Trp-l-Pro by attachment of a reverse dimethylallyl moiety to position C-2 of **3**, **4**, and **6**, respectively. This is expected for the EchPT1 reactions and also confirmed by intensive interpretation of their ^13^C, ^1^H-^1^H COSY, HSQC, and HMBC spectra. In the ^13^C NMR spectra of **7**, **8**, and **10**, the signals of C-2 at the indole ring as well as those of C-1˝, C-2˝, C-3˝, and C-4˝/C-5˝ of the reverse prenyl residue were observed at δ_c_ 141.1–141.8, 39.1–39.2, 145.8–145.9, 112.8–112.9, and 27.9–28.5 ppm, respectively (Figs. S11, S16, and S25). Inspection of the ^1^H and ^13^C NMR spectra of compound **9** revealed the presence of the signals for H-2 at δ_H_ 7.11 ppm and for C-2 at δ_c_ 123.9 ppm, suggesting that the additional prenyl residue is attached to another position than C-2. Remarkably, the signal of C-1˝ in **9** at δ_c_ 59.1 ppm was downfield shifted for approximate 20 ppm, in comparison to those of **7**, **8**, and **10**. This indicates its attachment to a hetero such as nitrogen than carbon atom (Fig. S20). Correspondingly, the signal of NH-1 was disappeared in the ^1^H NMR of **9**. Together with HSQC and HMBC data (Table S4), compound **9** was proven to be *N1,C6*-diprenylated derivative (Fig. 5).

### Determination of the kinetic parameters of EchPT1 toward monopernylated cyclo-L-Trp-L-Pro

**Fig. 6:**
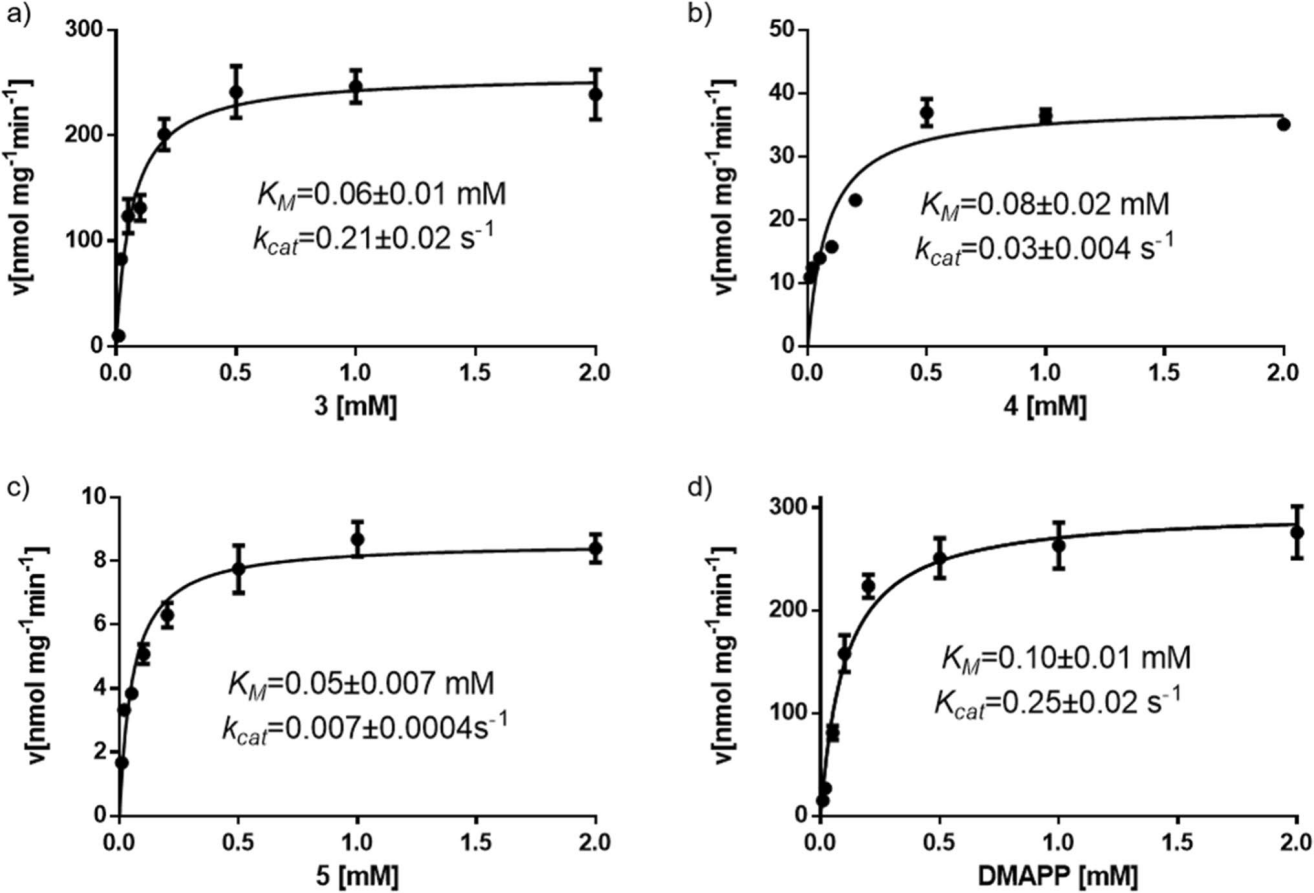
Determination of kinetic parameters of EchPT1 toward **3**–**5** and DMAPP with **3**

To better understand the behavior of EchPT1, kinetic parameters including Michaelis–Menten constant *K*_*M*_ and turnover number *k*_*cat*_ were determined for the three aromatic substrates **3**–**5** and DMAPP in the presence of the best accepted **3**. Kinetic parameters for **6** could not be obtained due to the impurity with **5**. All the reactions followed the Michaelis–Menten kinetics (Fig. 6). Similar affinities with *K*_*M*_ values between 0.05 and 0.08 mM were determined for **3**–**5**, comparable to that of *cyclo*-l-Trp-l-Ala, the natural substrate of EchPT1 at 0.09 mM (Wohlgemuth et al. 2017). The highest *k*_*cat*_ at 0.21 s^−1^ was calculated for **3**, followed by those of **4** and **5** at 0.03 and 0.007 s^−1^, respectively. These values are much lower than that of *cyclo*-l-Trp-l-Ala at 6.63 s^−1^ (Wohlgemuth et al. 2017). The catalytic efficiencies were calculated for **3**, **4**, and **5** to be 3500, 375, and 140 s^−1^ mM^−1^, respectively, being in good consistence with the observed conversion yields depicted in Fig. 5. The *K*_*M*_ value for DMAPP was determined in the presence of the best *C4*-prenylated *cyclo*-l-Trp-l-Pro **3** at 0.10 mM, slightly lower than in the presence of *cyclo*-l-Trp-l-Ala at 0.18 mM (Wohlgemuth et al. 2017) (Table 1).

**Table 1:** Kinetic parameters of EchPT1 toward **3**–**5** and DMAPP

Substrates	*K*_*M*_ (mM)	*k*_*cat*_ (s^−1^)	*k*_*cat*_/*K*_*M*_ (s^−1^ M^−1^)
**3**	0.06 ± 0.01	0.21 ± 0.02	3500
**4**	0.08 ± 0.02	0.03 ± 0.004	375
**5**	0.05 ± 0.007	0.007 ± 0.0004	140
DMAPP with **3**	0.10 ± 0.01	0.25 ± 0.02	2500

Three independent experiments were carried out and standard deviations are given as ± values

## Discussion

In nature, cyclodipeptides are assembled by nonribosomal peptide synthases, mostly in fungi, or by cyclodipeptide synthases, mainly in bacteria (Canu et al. 2020; Mishra et al. 2017). They are then modified by different tailoring enzymes such as prenyltransferases, methyltransferases, cytochrome P450 enzymes and 2-oxoglutarate-dependent monooxygenases (Harken and Li 2021; Winkelblech et al. 2015). Cyclodipeptide prenylation is usually initiated at position C-2 by reverse or regular prenyltransferases (Fig. 1) (Winkelblech et al. 2015). Further prenylations take place thereafter at the benzene ring, as demonstrated in the biosynthesis of the triprenylated *cyclo*-l-Trp-l-Ala derivative echinulin (Wohlgemuth et al. 2017).

In this study, we first followed the nature´s biosynthetic strategy for production of diprenylated *cyclo*-l-Trp-l-Pro, *i.e*., at *C2*,*C4*-, *C2*,*C5*-, *C2*,*C6*-, and *C2*,*C7*-diprenylated derivatives. We first prepared deoxybrevianamide E by prenylation of *cyclo*-l-Trp-l-Pro at position C-2 with EchPT1. Unfortunately, the four prenyltransferases FgaPT2_R244L, FgaPT2_Y398F, 6-DMATS_Sa_, and 7-DMATS used in this study showed strict substrate specificity and did not accept deoxybrevianamide E as substrate for further prenylation (Fig. 3). We therefore changed our strategy by first prenylation of *cyclo*-l-Trp-l-Pro at the benzene ring with the four enzymes FgaPT2_R244L, FgaPT2_Y398F, 6-DMATS_Sa_, and 7-DMATS. As expected, three different monoprenylated derivatives, i.e., *cyclo*-l-Trp-l-Pro with dimethylally moieties at the positions C-4, C-5, and C-7, were then successfully converted by EchPT1 to the designed diprenylated products (Fig. 5). However, to our surprise, EchPT1 catalyzed the unique *N1*-reverse prenylation of *C6*-prenylated *cyclo*-l-Trp-l-Pro and produced *N1*,*C6*-diprenylated product. This is the first example for an *N1*-prenylation catalyzed by EchPT1. This study expands therefore significantly our knowledge on prenyltransferases of the DMATS superfamily. It is obvious that their catalytic potential is far away from exhausted, even a large number of biochemical works have been carried out and published in the last decade (Fan et al. 2015; Mori 2020). Furthermore, our results provide a new strategy for production of designed products and increasing structural diversity by changing the normal reaction sequences.

## Supplementary Information

Below is the link to the electronic supplementary material.

Supplementary file1 (PDF 4.12 MB)

## Data Availability

All data generated during this study are included in this published article and its supplementary information file.
