# Supplementary material for: Diprenylated cyclodipeptide production by changing the prenylation sequence of the nature’s synthetic machinery
Source: Appl Microbiol Biotechnol. 2022 Nov 28;107(1):261–71. doi: 10.1007/s00253-022-12303-4 (PMC9750918; doi:10.1007/s00253-022-12303-4)
Supplement: Supplementary file 1 — Supplementary file1 (PDF 4.12 MB) [file 253_2022_12303_MOESM1_ESM.pdf]

Supporting Information

Diprenylated Cyclodipeptide Production by Changing the Prenylation Sequence of the Nature´s Synthetic Machinery

Wen Li, Lindsay Coby, Jing Zhou, Shu-Ming Li*

Institut für Pharmazeutische Biologie und Biotechnologie, Fachbereich Pharmazie, Philipps-Universität Marburg, Robert-Koch Straße 4, 35037 Marburg, Germany

Corresponding to Shu-Ming Li, E-mail: shuming.li@staff.uni-marburg.de

Table of Contents

Supplementary Tables 3

Table S1 NMR data of compounds **2**–**4** in $CDCl_3$ 3

Table S2 NMR data of compounds **5** and **6** in $CDCl_3$ 4

Table S3 NMR data of compounds **7** and **8** in $CDCl_3$ 5

Table S4 NMR data of compounds **9** and **10** in $CDCl_3$ 6

Supplementary Figures 7

Fig. S1 ^1H NMR spectrum of **2** in $CDCl_3$ (500 MHz). 7

Fig. S2 ^1H NMR spectrum of **3** in $CDCl_3$ (500 MHz). 7

Fig. S3 ^1H NMR spectrum of **4** in $CDCl_3$ (500 MHz). 8

Fig. S4 ^1H NMR spectrum of **5** in $CDCl_3$ (500 MHz). 8

Fig. S5 ^{13}C NMR spectrum of **5** in $CDCl_3$ (125 MHz). 9

Fig. S6 ^1H-^1H COSY spectrum of **5** in $CDCl_3$. 9

Fig. S7 HSQC spectrum of **5** in $CDCl_3$. 10

Fig. S8 HMBC spectrum of **5** in $CDCl_3$ 10

Fig. S9 ^1H NMR spectrum of **6** (mixture with **5**) in $CDCl_3$ (500 MHz). 11

Fig. S10 ^1H NMR spectrum of **7** in $CDCl_3$ (500 MHz). 11

Fig. S11 ^{13}C NMR spectrum of **7** in $CDCl_3$ (125 MHz). 12

Fig. S12 ^1H-^1H COSY spectrum of **7** in $CDCl_3$. 12

Fig. S13 HSQC spectrum of **7** in $CDCl_3$. 13

Fig. S14 HMBC spectrum of **7** in $CDCl_3$. 13

Fig. S15 ^1H NMR spectrum of **8** in $CDCl_3$ (500 MHz). 14

Fig. S16 ^{13}C NMR spectrum of **8** in $CDCl_3$ (125 MHz). 14

Fig. S17 ^1H-^1H COSY spectrum of **8** in $CDCl_3$. 15

Fig. S18 HSQC spectrum of **8** in $CDCl_3$. 15

Fig. S19 HMBC spectrum of **8** in $CDCl_3$. 16

Fig. S20 ^1H NMR spectrum of **9** in $CDCl_3$ (500 MHz). 16

Fig. S21 ^{13}C NMR spectrum of **9** in $CDCl_3$ (125 MHz). 17

Fig. S22 HSQC spectrum of **9** in $CDCl_3$. 17

Fig. S23 HMBC spectrum of **9** in $CDCl_3$. 18

Fig. S24 ^1H NMR spectrum of **10** in $CDCl_3$ (500 MHz). 18

Fig. S25 ^{13}C NMR spectrum of **10** in $CDCl_3$ (125 MHz). 19

References 20

Supplementary Tables

Table S1 NMR data of compounds **2**–**4** in $CDCl_3$

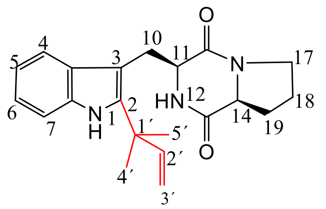

2

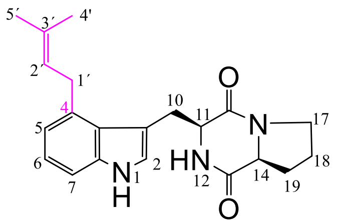

3

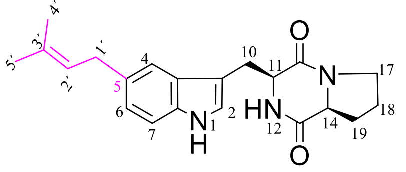

4

Pos.	δ_H (ppm) multi. J (Hz)	δ_H (ppm) multi. J (Hz)	δ_H (ppm) multi. J (Hz)
1	8.07 br s	8.25 br s	8.06 br s
2	-	7.07 s	7.08 d (1.6)
4	7.49 d (8.0)	-	7.35 s
5	7.11 ddd (8.0, 7.1, 1.4)	6.93 d (7.2)	-
6	7.18 ddd (8.0, 7.1, 1.4)	7.14 t (7.4)	7.07 dd (8.3, 1.6)
7	7.33 dd (8.0, 1.4)	7.24 d (8.0)	7.31 d (8.3)
10a	3.75 dd (15.5, 4.3)	3.98 dd (15.4, 3.0)	3.76 dd (15.0, 3.5)
10b	3.19 dd (15.5, 11.7)	2.98 dd (15.4, 11.3)	2.92 dd (15.0, 11.3)
11	4.44 dd (11.7, 4.3)	4.29 dd (11.3, 3.0)	4.37 dd (11.3, 3.5)
12	5.69 br s	5.84 br s	5.72 br s
14	4.07 t (7.7)	4.09 t (8.3)	4.09 t (8.2)
17	3.68 ddd (12.0, 9.5, 3.1)	3.67[a] m	3.67 dt (11.9, 8.5)
	3.60 ddd (12.0, 9.5, 3.1)	3.61 ddd (11.8, 8.9, 3.1)	3.60 ddd (11.8, 8.5, 3.1)
18	2.08 m	2.08 m	2.04 m
	1.92 m	1.92 m	1.91 m
19	2.35 m	2.35 m	2.34 m
	2.08 m	2.08 m	2.04 m
1′	-	3.75 dd (16.1, 6.8)	3.43 d (7.2)
	-	3.67[a] m	
2′	6.14 dd (17.7, 10.4)	5.32 tsept (6.8, 1.4)	5.37 tsept (7.2, 1.4)
3′	5.20 dd (17.7, 0.8)	-	-
	5.18 dd (10.4, 0.8)	-	-
4′	1.56 s	1.74 br s	1.75 br s
5′	1.56 s	1.74 br s	1.75 br s

[a] Signals overlapping with each other.

The NMR data of **2** and **3** correspond well to those published previously (Schkeryantz et al. 1999; Steffan and Li 2009).

Table S2 NMR data of compounds **5** and **6** in $CDCl_3$

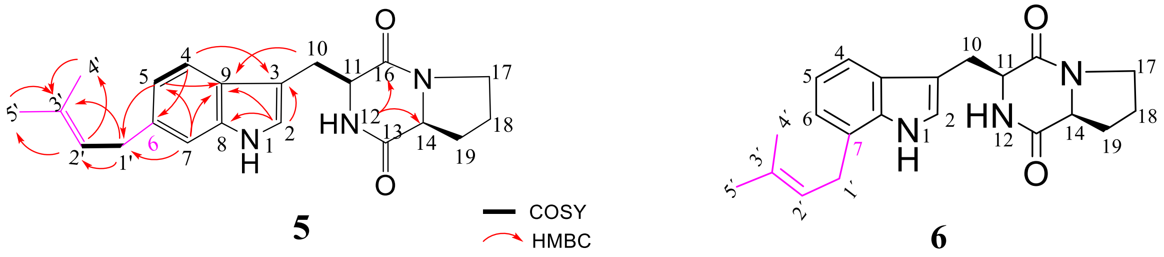

Pos.	δ_H (ppm) multi. *J* (Hz)	δ_C	HMBC	δ_H (ppm) multi. *J* (Hz)
1	8.23 br s	-		8.13 br s
2	7.05 s	123.1	C-3, C-8, C-9	7.10 s
3	-	109.9		-
4	7.49 d (8.1)	118.5	C-3, C-6	7.44 d (7.6)
5	6.99 d (8.1)	121.3	C-7, C-9, C-1′	7.07 t (7.7)
6	-	137.0		7.04 d (7.7)
7	7.20 br s	110.9	C-5, C-9, C-1′	-
8	-	137.3		-
9	-	125.0		-
10a	3.75 dd (15.0, 3.6)	27.1	C-9	3.76 dd (15.0, 3.8)
10b	2.92 dd (15.0, 11.1)			2.93 dd (15.0, 11.1)
11	4.36 dd (11.1, 3.6)	54.7		4.37 dd (11.1, 3.8)
12	5.79 br s	-	C-14, C-16	5.71 br s
13	-	169.5		-
14	4.07 t (8.0)	59.4	C-12	4.07 t (8.0)
16	-	165.7		-
17	3.66 dt (11.9, 8.5)	45.6		3.65 dt (11.8, 8.5)
	3.59 ddd (11.9, 8.5, 3.2)			3.60 ddd (11.8, 8.5, 3.1)
18	2.03 m	22.8		2.03 m
	1.91 m			1.91 m
19	2.33 m	28.5		2.34 m
	2.03 m			2.03 m
1′	3.45 d (7.2)	34.7	C-5, C-6, C-7, C-2′, C-3′	3.57 d (7.1)
2′	5.38 t (7.2)	123.9	C-1′, C-4′, C-5′	5.38 tsept (7.1, 1.4)
3′	-	132.4		-
4′	1.76 br s	18.0	C-2′, C-3′	1.79 br s
5′	1.75 br s	26.0	C-2′, C-3′	1.83 br s

Note: Compound **5** was isolated as the sole product from the enzyme assay of **1** with 6-DMATS$_{Sa}$. Both **5** and **6** were isolated as a mixture in a ratio of 0.6:1 from the reaction mixture of **1** with 7-DMATS.

The NMR data of **6** correspond to those of *cyclo*-L-7-dimethylallyl-Trp-L-Pro reported previously (Liu et al. 2020).

Table S3 NMR data of compounds **7** and **8** in $CDCl_3$

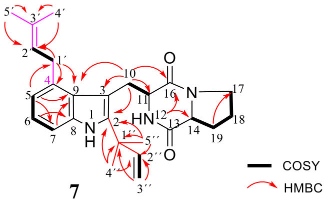

7

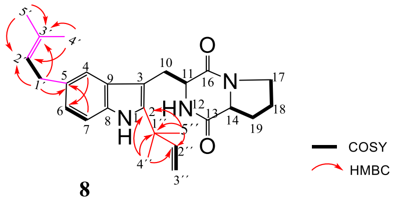

8

Pos.	δ_H (ppm) multi. *J* (Hz)	δ_C	HMBC	δ_H (ppm) multi. *J* (Hz)	δ_C	HMBC
1	8.12 br s	-		7.96 br s	-	
2	-	141.5[c]		-	141.8	C-4″, C-5″
3	-	105.4		-	104.4	
4	-	133.9		7.25 br s	117.1	C-6
5	6.89 d (7.2)	121.5	C-7, C-9, C-1′	-	133.8	
6	7.08 t (7.7)	122.4	C-8	7.00 dd (8.3, 1.4)	123.2	
7	7.18 d (8.0)	109.1	C-5, C-9	7.23 d (8.3)	110.9	C-5
8	-	135.2		-	133.0	
9	-	126.9[c]		-	129.4	
10a	3.78 dd (15.7, 4.3)	26.5	C-2, C-3, C-9, C-11, C-16	3.72 dd (15.3, 4.2)	26.1	
10b	3.41 dd (15.7, 11.3)			3.17 dd (15.3, 11.2)		
11	4.35 dd (11.3, 4.3)	55.7[c]		4.44 dd (11.2, 4.2)	54.9	
12	5.66 br s	-	C-14, C-16	5.72 br s	-	
13	-	169.2		-	169.3	
14	4.04 t (7.9)	59.4		4.08 t (8.0)	59.4	
16	-	166.0		-	166.1	
17	3.67[a] m	45.4		3.67 m	45.5	
	3.58 ddd (11.6, 9.0, 2.9)			3.60 ddd (11.8, 8.9, 3.1)		
18	2.06[b] m	22.8		2.08 m	22.8	
	1.92 m			1.92 m		
19	2.35 m	28.5	C-17	2.36 m	28.5	
	2.06[b] m			2.08 m		
1′	3.67[a] m	32.3	C-4, C-5, C-9, C-2′	3.41 d (7.2)	34.8	C-5, C-2′, C-3′
2′	5.28 tsept (6.8, 1.4)	123.7		5.35 tsept (7.2, 1.4)	124.5	C-1′, C-4′, C-5′
3′	-	132.7		-	131.9	
4′	1.72 br s	18.2	C-2′, C-3′	1.74 br s	18.1	C-2′, C-3′
5′	1.69 br s	25.9	C-2′, C-3′	1.73 br s	26.0	C-2′, C-3′
1″	-	39.2		-	39.2	
2″	6.14 dd (17.4, 10.5)	145.9	C-1″, C-4″, C-5″	6.12 dd (17.5, 10.5)	145.8	C-4″, C-5″
3″	5.19 dd (17.4, 0.6)	112.8	C-1″, C-2″	5.17 d (17.5)	112.9	
	5.16 dd (10.5, 0.6)			5.18 d (10.5)		
4″	1.57 s	28.2	C-2, C-1″, C-2″	1.55 s	28.5	C-2
5″	1.56 s	28.1	C-2, C-1″, C-2″	1.54 s	28.1	C-2

[a, b] Signals overlapping with each other; [c] Signals were only detected in HSQC.

Table S4 NMR data of compounds **9** and **10** in $CDCl_3$

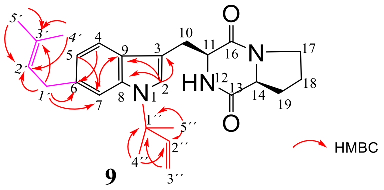

9

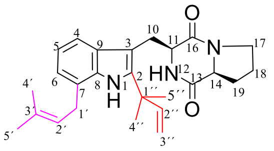

10

Pos.	δ_H (ppm) multi. *J* (Hz)	δ_C	HMBC	δ_H (ppm) multi. *J* (Hz)	δ_C
1	-	-		8.13 br s	-
2	7.11 br s	123.9	C-3, C-8, C-9	-	141.1
3	-	107.7[a]		-	104.7
4	7.45 d (8.1)	118.4	C-6	7.34 d (7.9)	116.1
5	6.94 dd (8.1, 1.3)	120.4	C-7	7.03 t (7.8)	120.4
6	-	135.4		6.97 dd (7.2, 0.8)	122.0
7	7.33 br s	113.5	C-5, C-9, C-1′	-	123.8
8	-	136.6		-	133.8
9	-	126.8		-	129.2
10	3.75 dd (15.0, 3.6)	27.1		3.72 dd (15.2, 4.1)	26.2
	2.87 dd (15.0, 11.3)			3.17 dd (15.2, 11.2)	
11	4.35 dd (11.3, 3.6)	54.8		4.44 dd (11.2, 4.1)	55.0
12	5.72 br s	-		5.69 br s	-
13	-	169.5		-	169.4
14	4.07 t (7.9)	59.3		4.05 t (8.4)	59.4
16	-	165.7		-	166.0
17	3.67 m	45.5		3.68 m	45.5
	3.59 ddd (11.7, 8.8, 2.9)			3.60 m	
18	2.03 m	22.7		2.07 m	22.8
	1.91 m			1.93 m	
19	2.34 m	28.4		2.34 m	28.5
	2.03 m			2.07 m	
1′	3.41 d (7.4)	34.8	C-6, C-7, C-2′, C-3′	3.57 d (7.4)	31.4
2′	5.36 tsept (7.4, 1.5)	124.0	C-1′, C-4′, C-5′	5.43 tsept (7.4, 1.5)	122.9
3′	-	132.3		-	133.2
4′	1.75 br s	17.9	C-2′, C-3′	1.88 br s	18.1
5′	1.75 br s	25.9	C-2′, C-3′	1.81 br s	25.9
1″	-	59.1		-	39.1
2″	6.13 dd (17.5, 10.7)	144.2	C-4″, C-5″	6.12 dd (17.6, 10.4)	145.8
3″	5.20 dd (17.5, 0.5)	113.6	C-4″, C-5″	5.18 dd (17.6, 0.8)	112.8
	5.23 dd (10.7, 0.5)			5.17 dd (10.4, 0.8)	
4″	1.74 s	27.9	C-1″, C-2″	1.55 s	28.0
5″	1.73 s	27.8	C-1″, C-2″	1.53 s	27.9

[a] Signal was only detected in HSQC.

Supplementary Figures

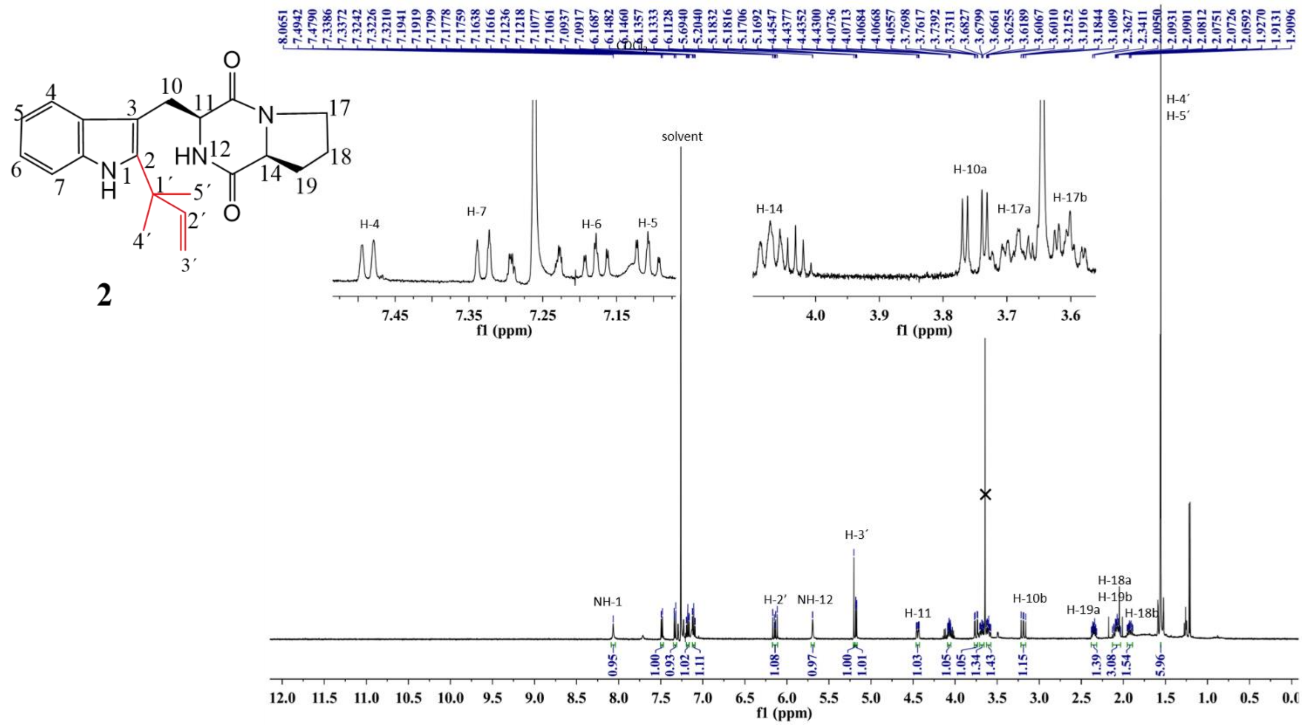

Fig. S1 ^{1}H NMR spectrum of **2** in $CDCl_3$ (500 MHz).

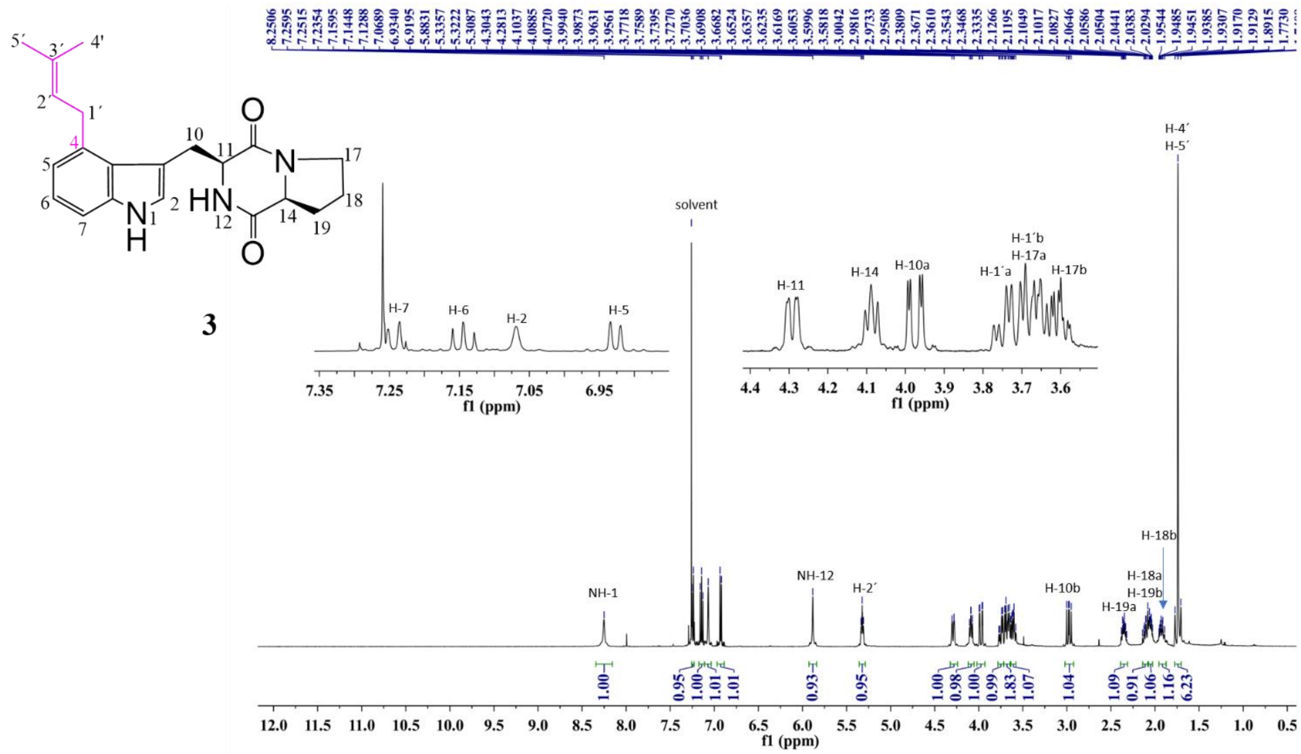

Fig. S2 ^{1}H NMR spectrum of **3** in $CDCl_3$ (500 MHz).

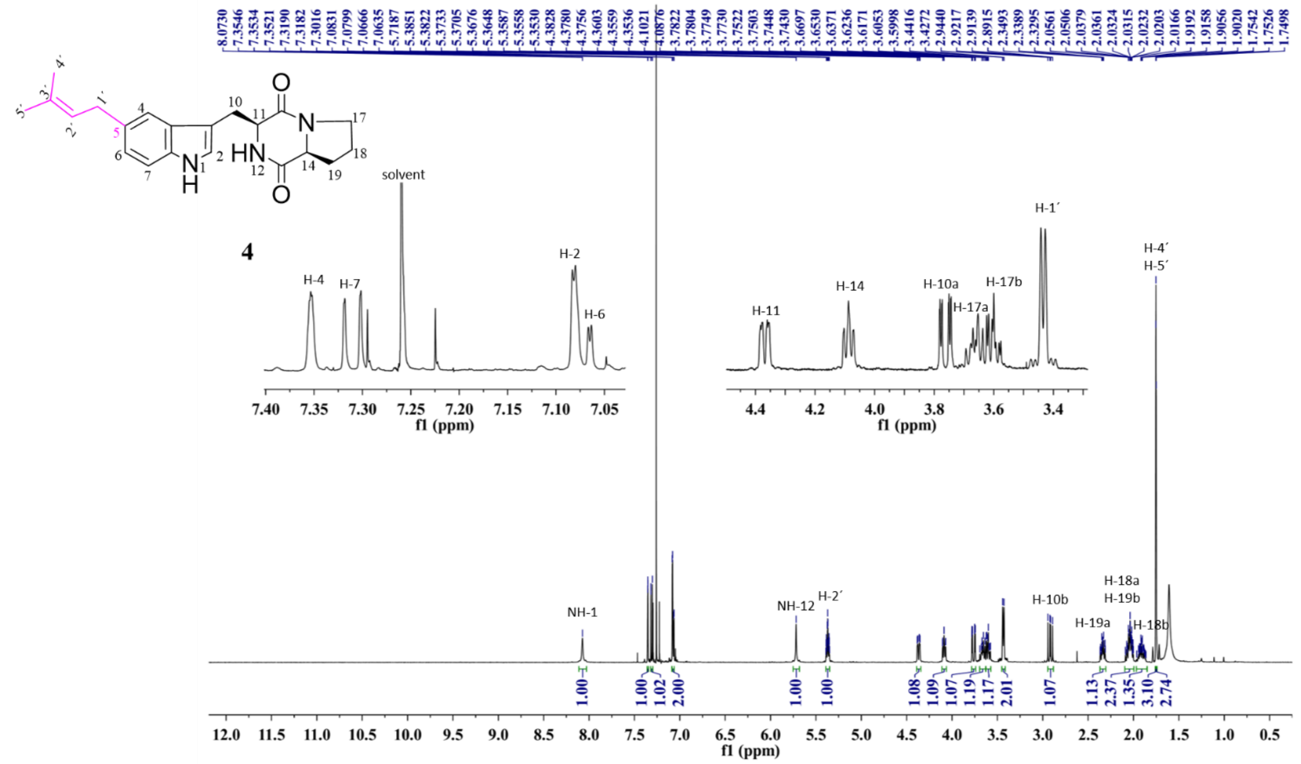

Fig. S3 ^{1}H NMR spectrum of **4** in $CDCl_3$ (500 MHz).

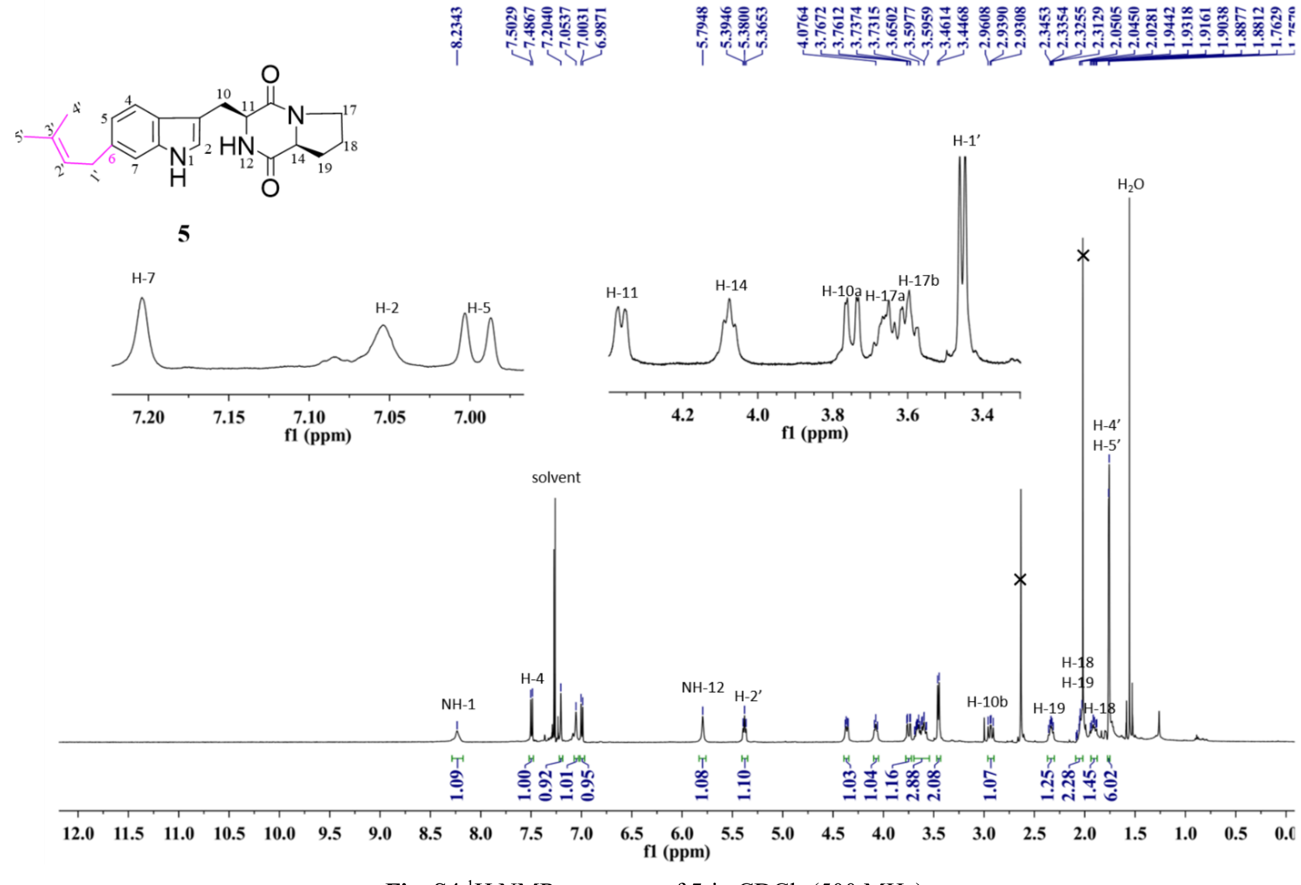

Fig. S4 ^{1}H NMR spectrum of **5** in $CDCl_3$ (500 MHz).

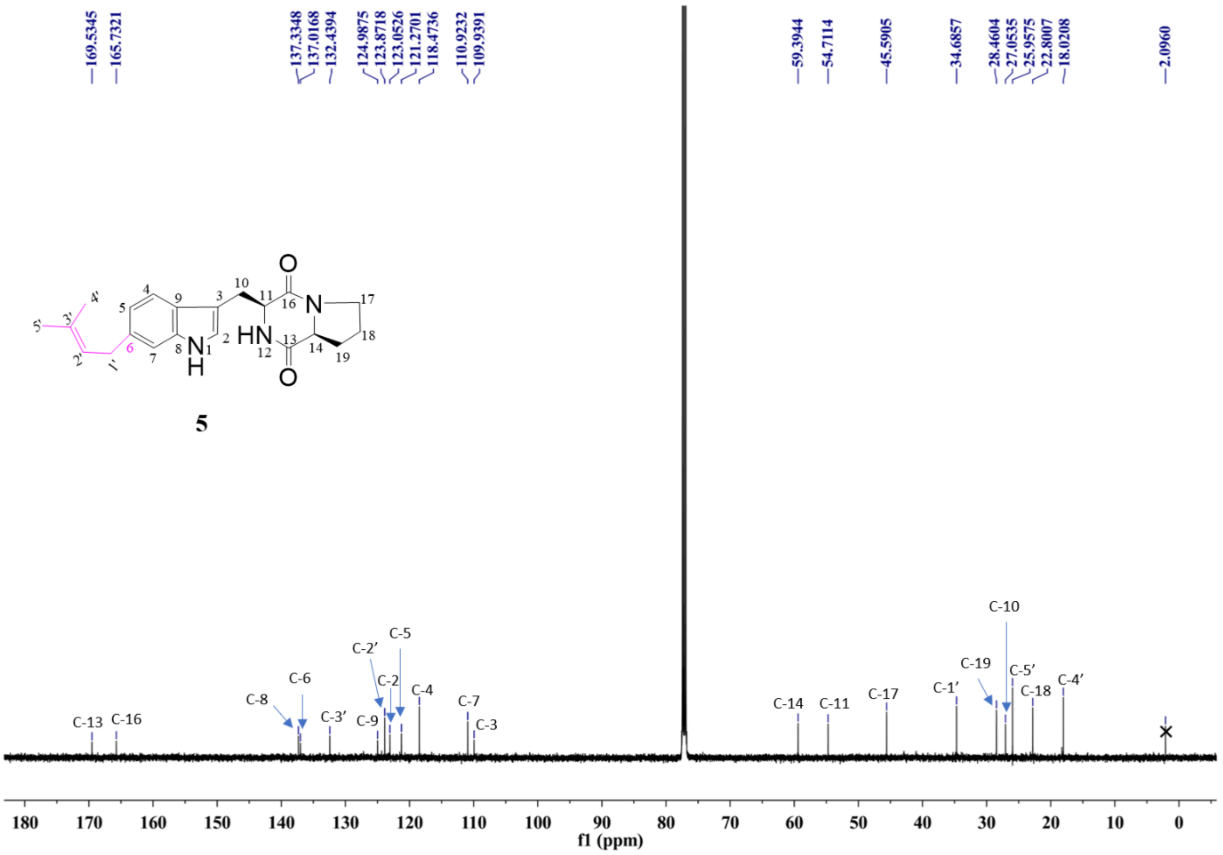

Fig. S5 ^{13}C NMR spectrum of **5** in $CDCl_3$ (125 MHz).

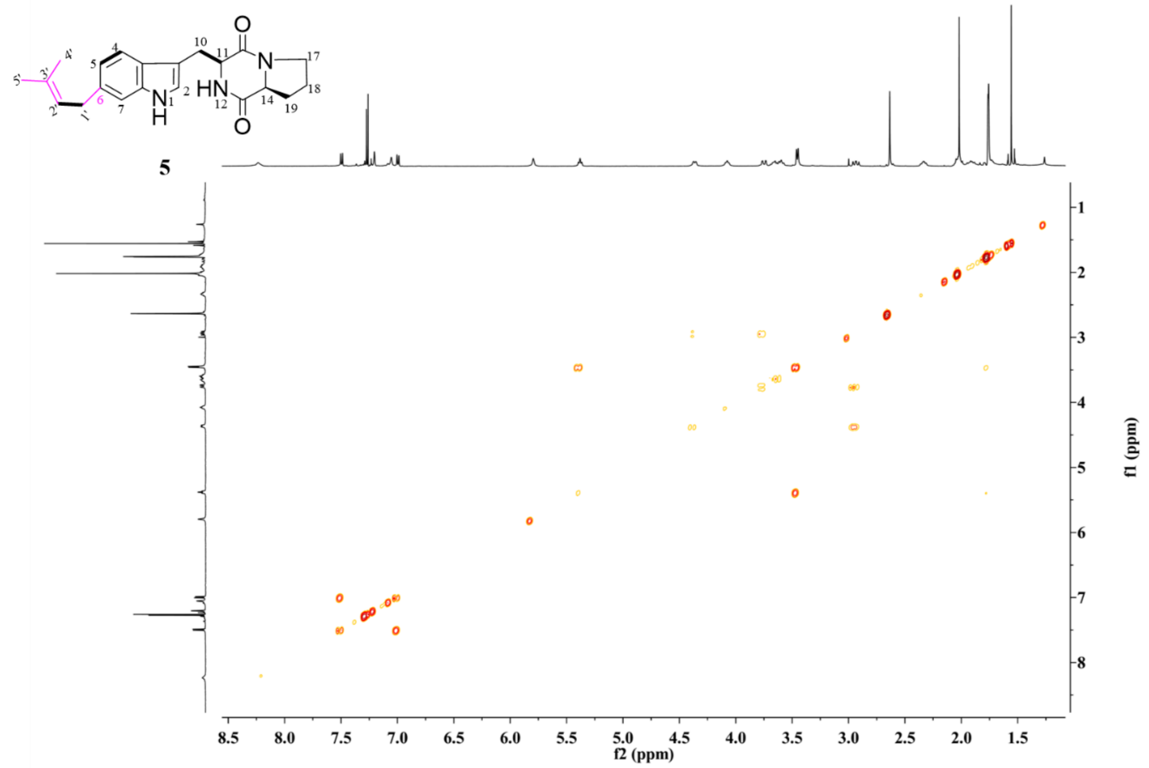

Fig. S6 ^{1}H-^{1}H COSY spectrum of **5** in $CDCl_3$.

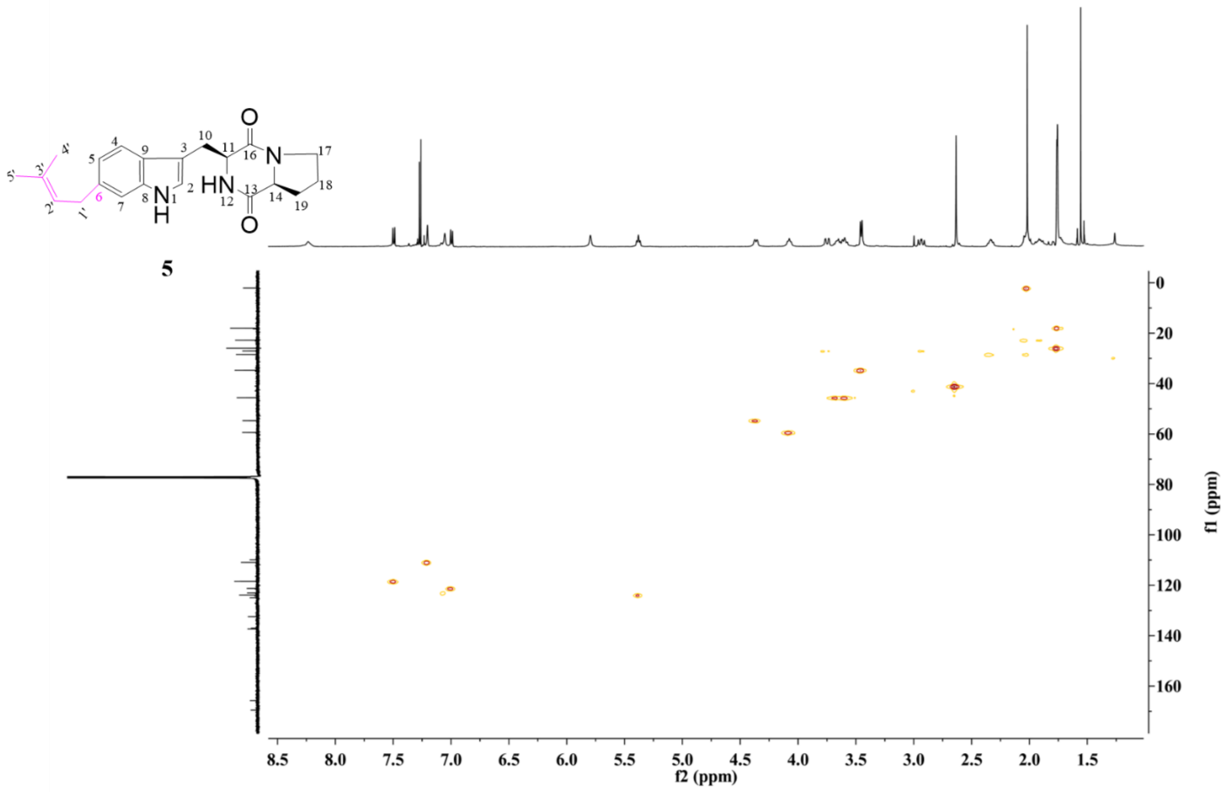

Fig. S7 HSQC spectrum of **5** in $CDCl_3$.

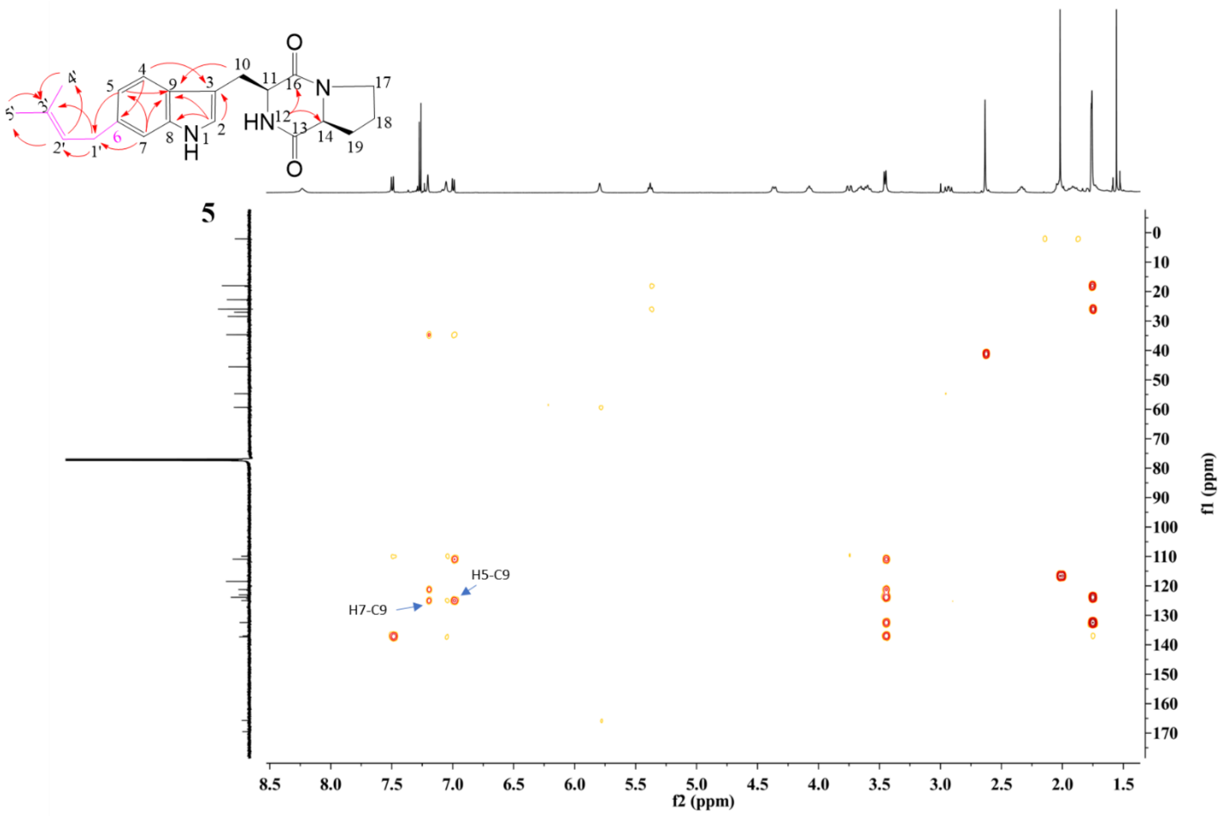

Fig. S8 HMBC spectrum of **5** in $CDCl_3$.

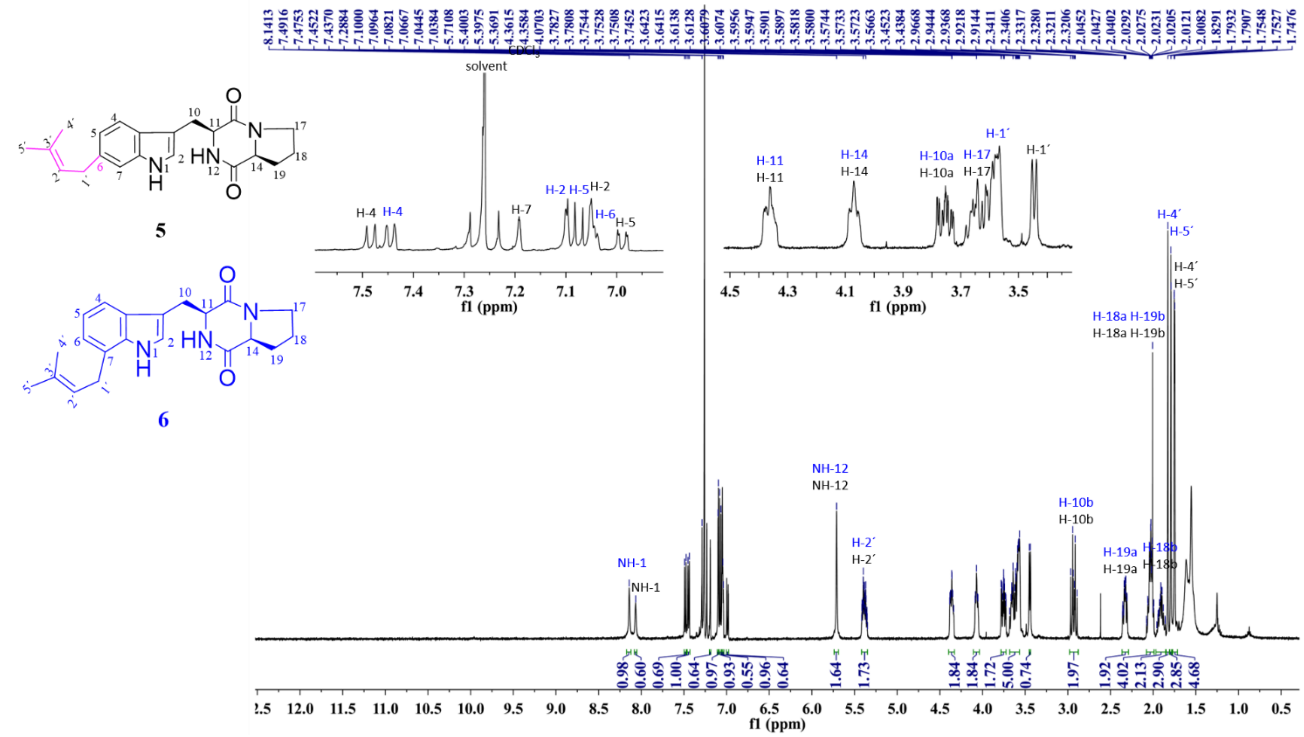

Fig. S9 ^{1}H NMR spectrum of **6** (mixture with **5**) in $CDCl_3$ (500 MHz).

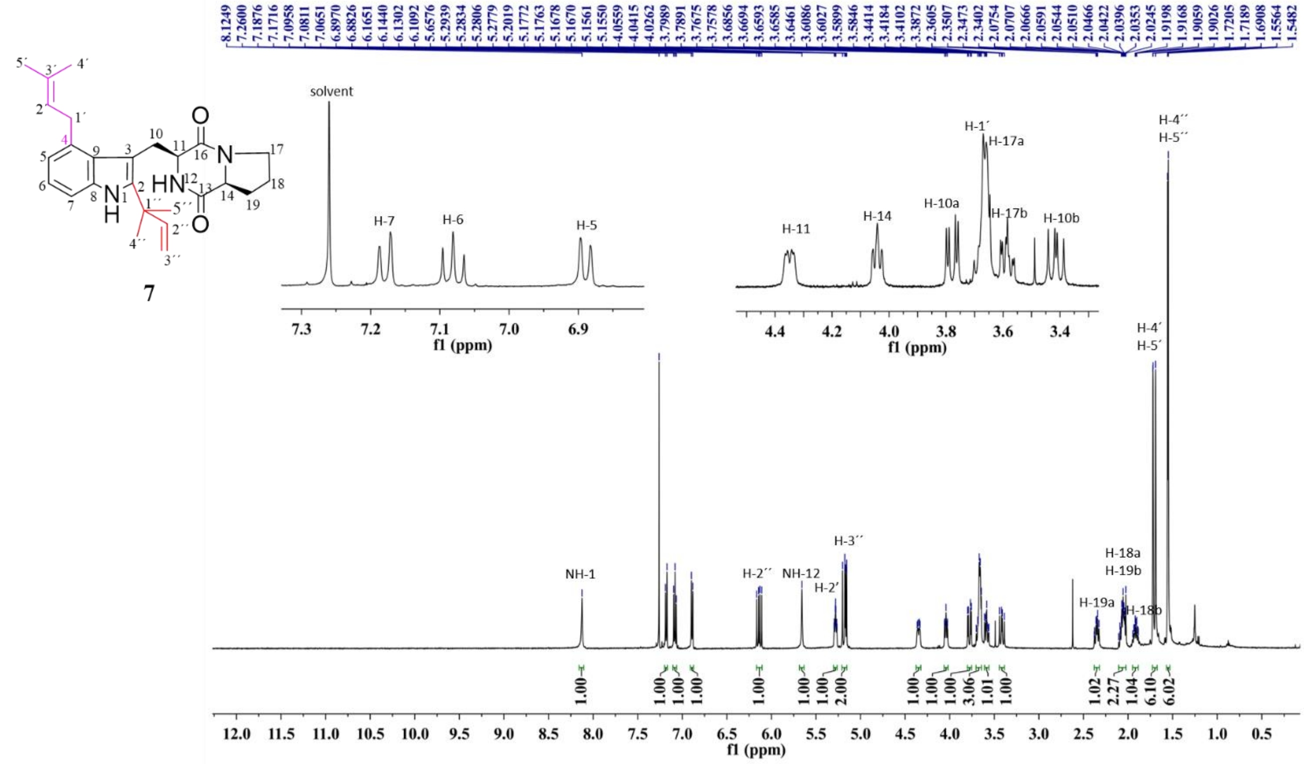

Fig. S10 ^{1}H NMR spectrum of **7** in $CDCl_3$ (500 MHz).

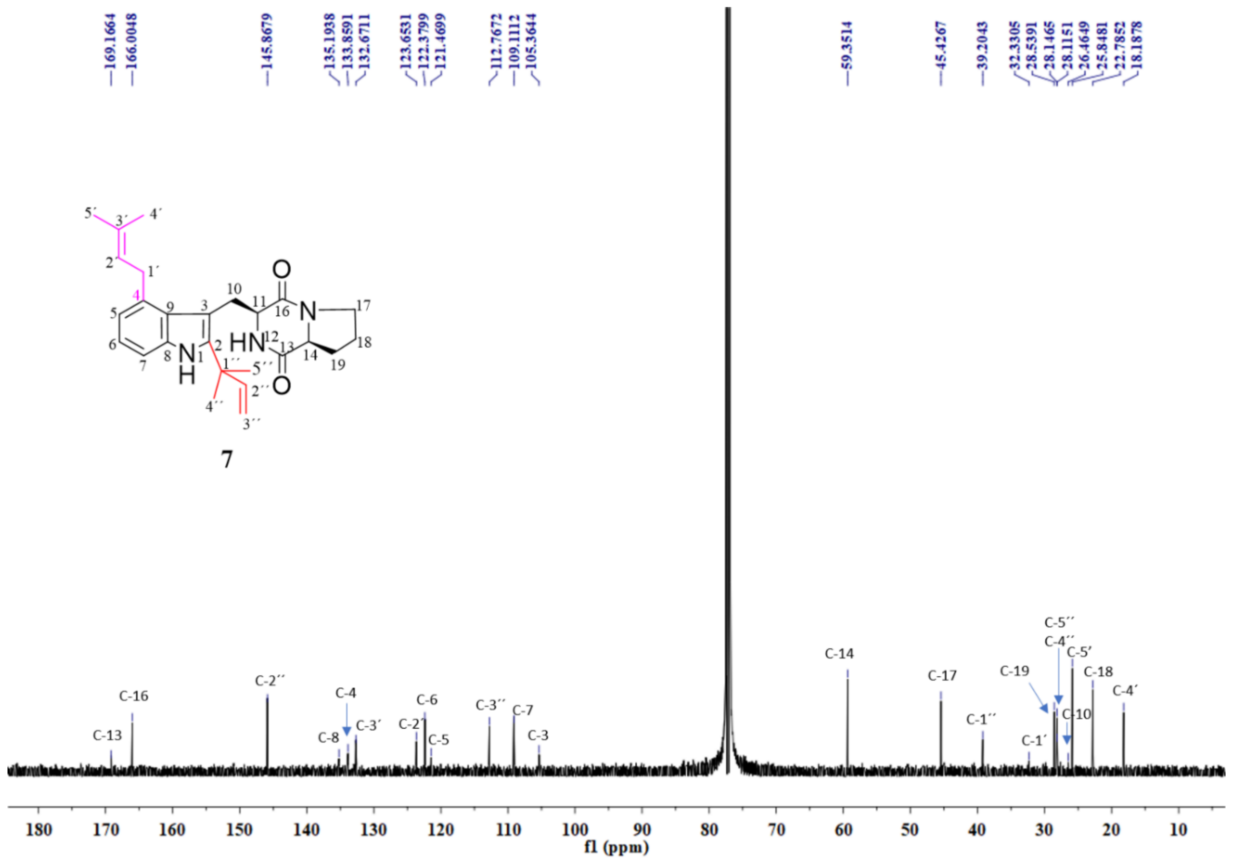

Fig. S11 ^{13}C NMR spectrum of **7** in $CDCl_3$ (125 MHz).

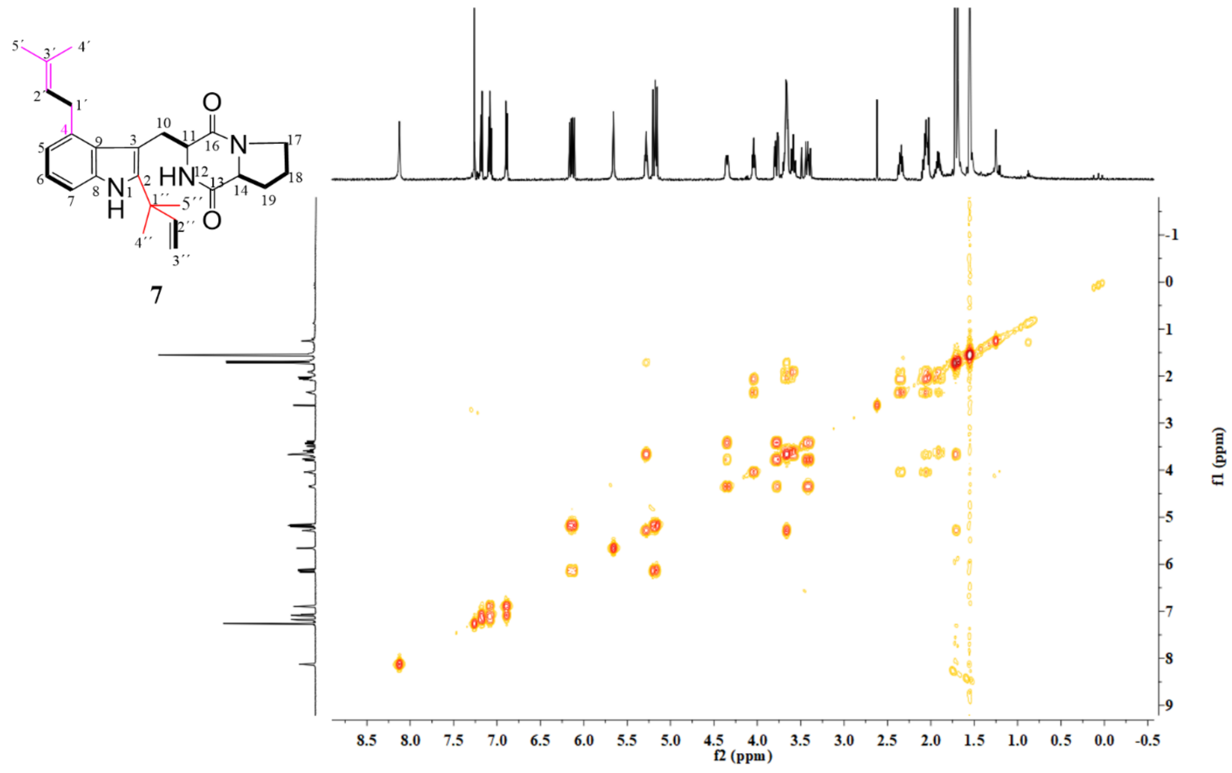

Fig. S12 ^{1}H-^{1}H COSY spectrum of **7** in $CDCl_3$.

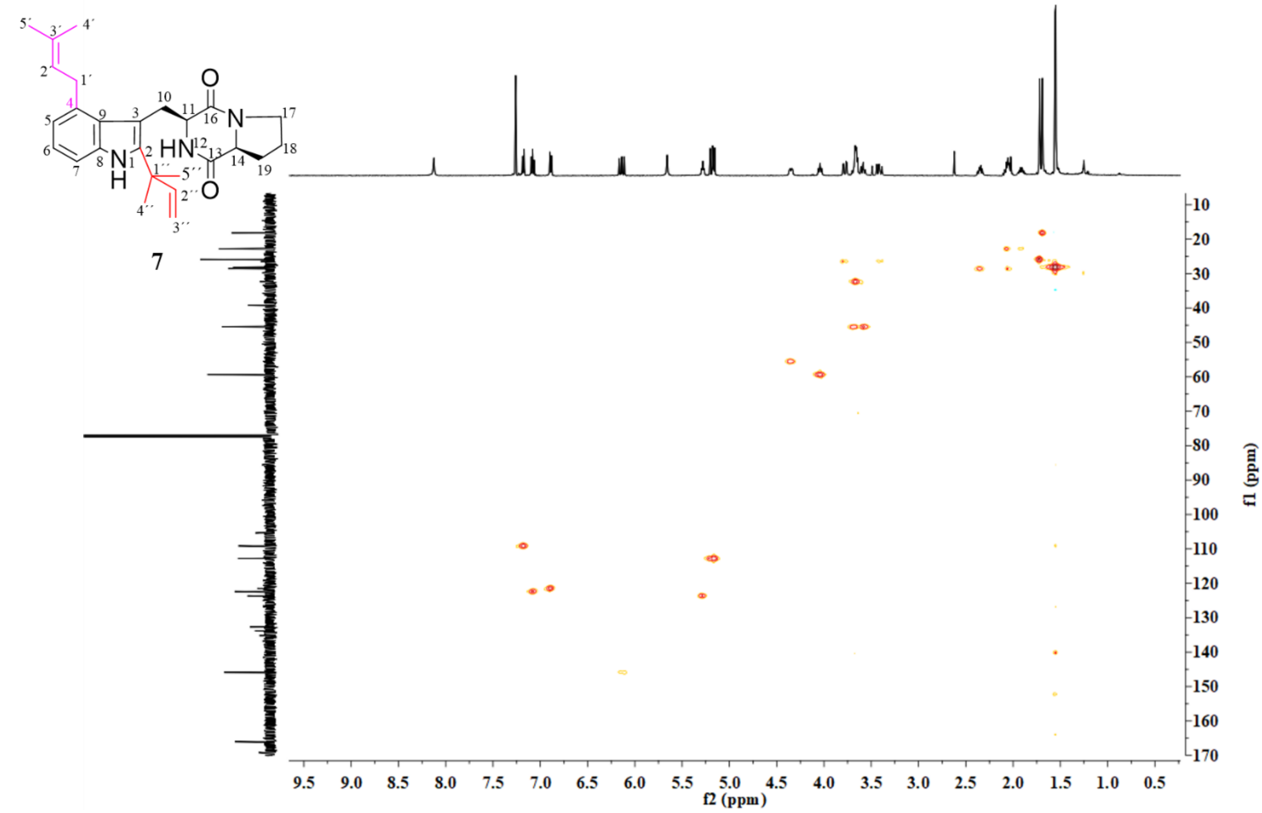

Fig. S13 HSQC spectrum of **7** in $CDCl_3$.

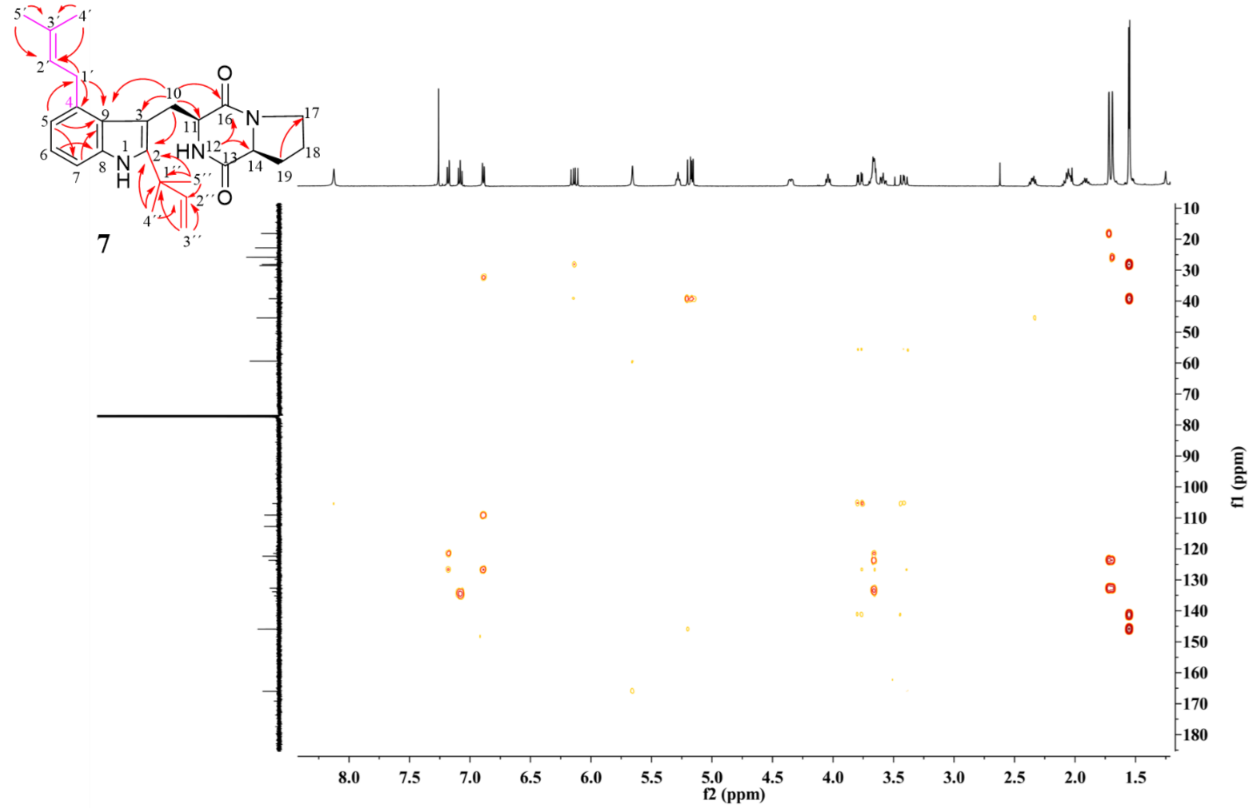

Fig. S14 HMBC spectrum of **7** in $CDCl_3$.

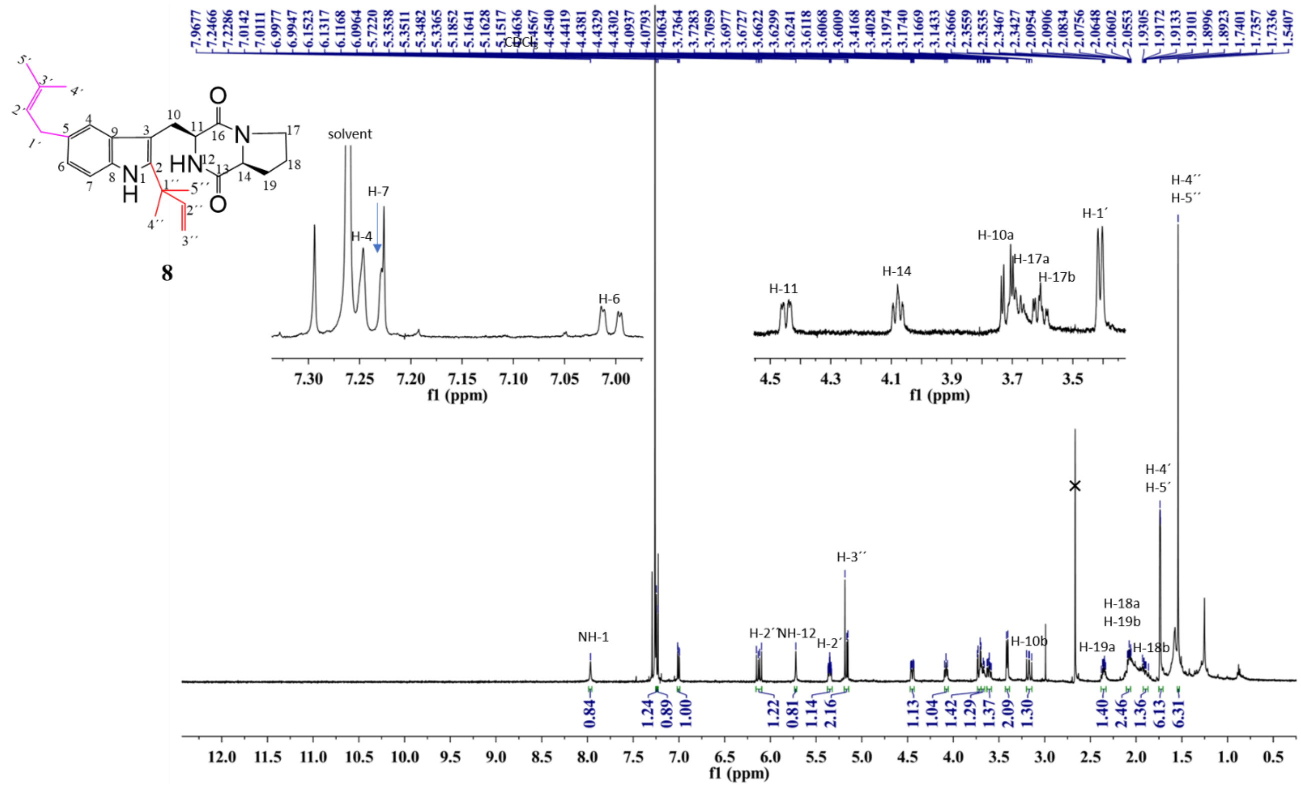

Fig. S15 ^{1}H NMR spectrum of **8** in $CDCl_3$ (500 MHz).

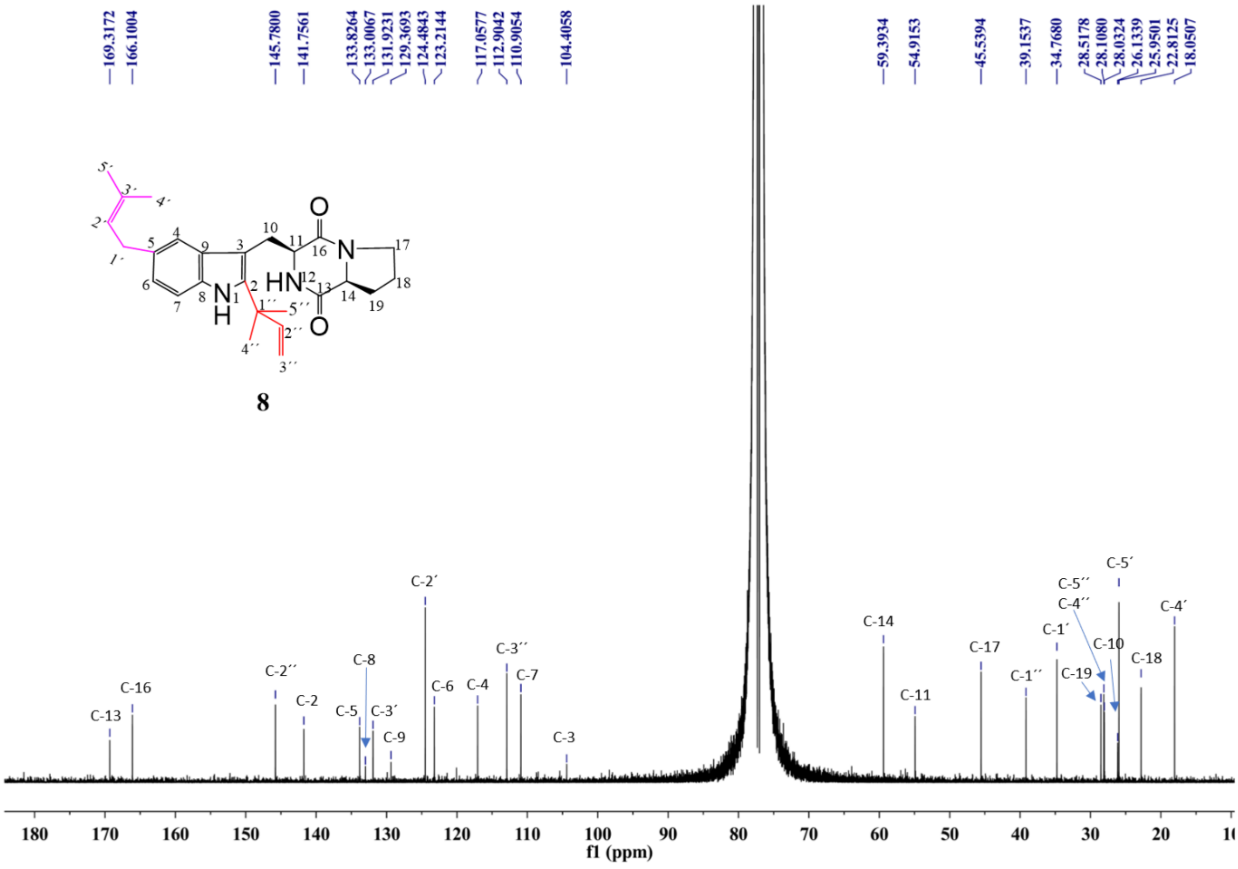

Fig. S16 ^{13}C NMR spectrum of **8** in $CDCl_3$ (125 MHz).

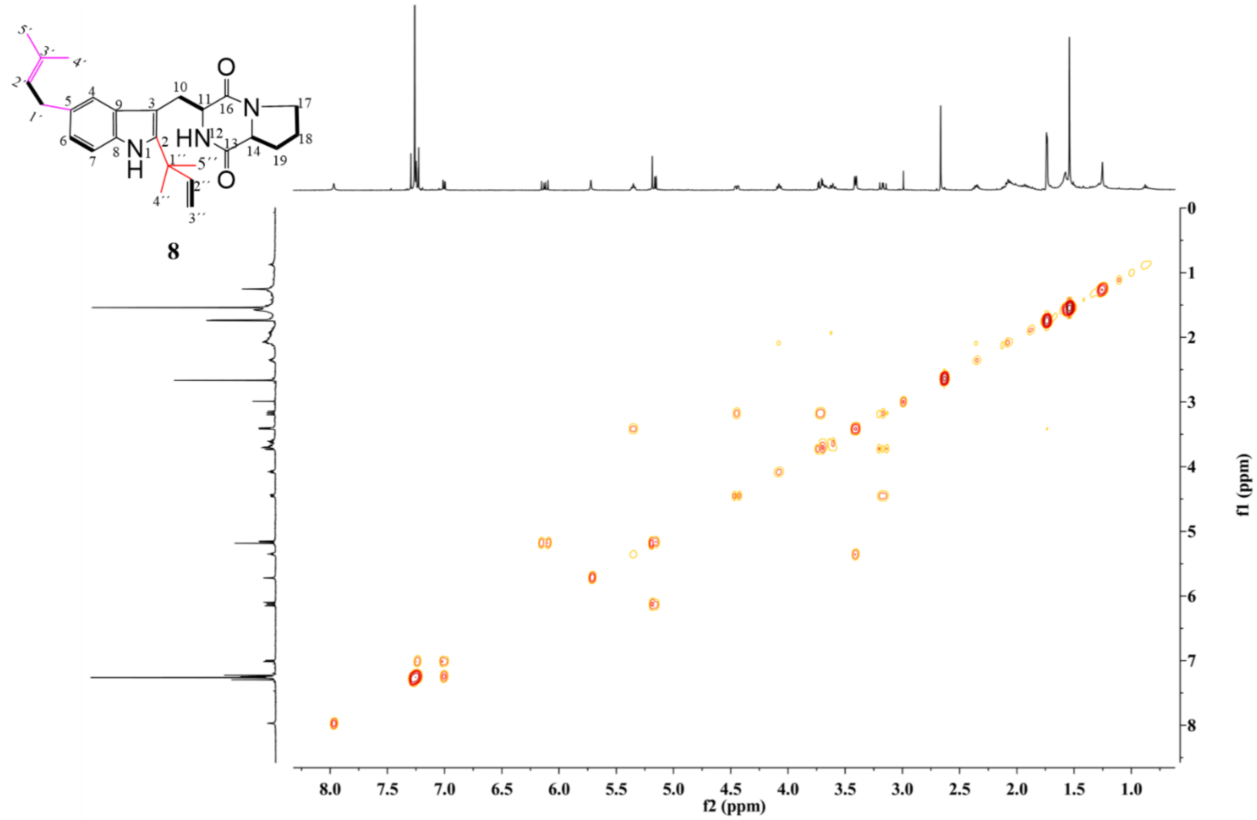

Fig. S17 ^{1}H-^{1}H COSY spectrum of **8** in $CDCl_3$.

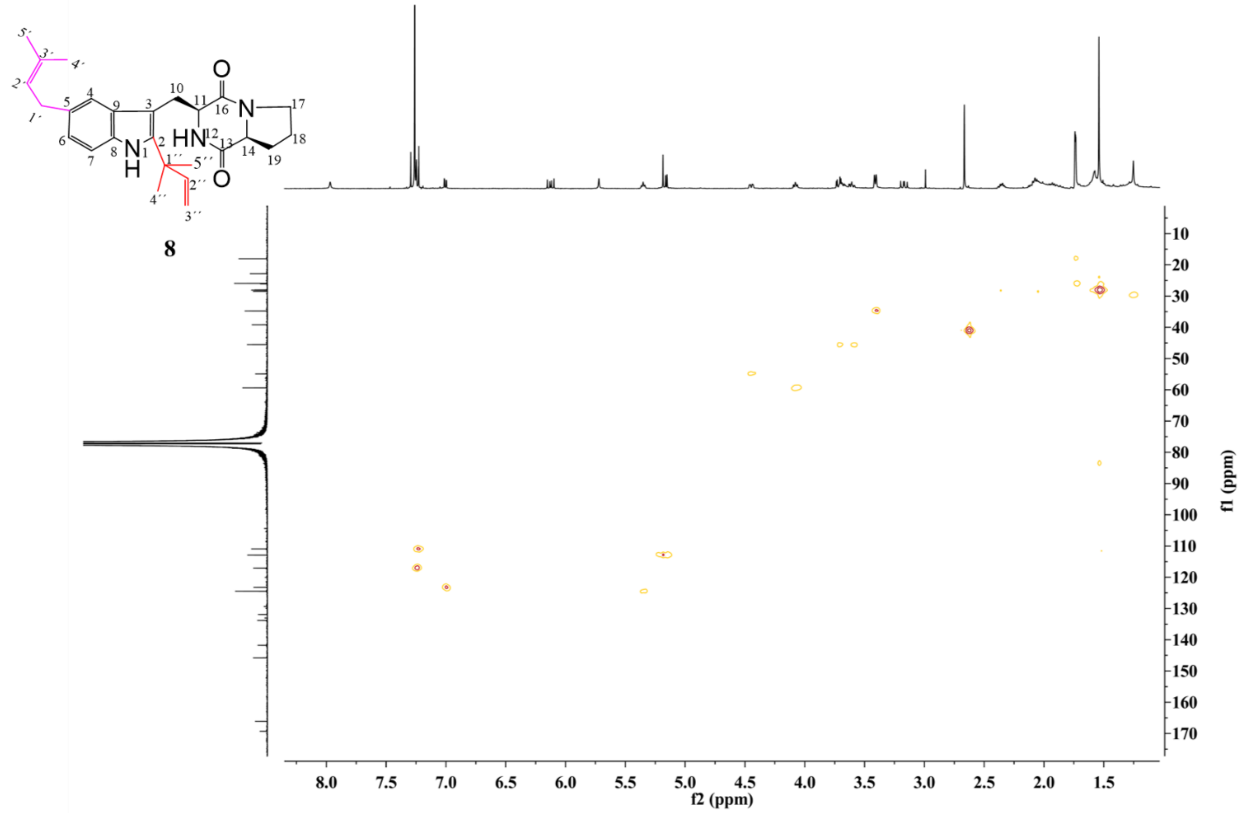

Fig. S18 HSQC spectrum of **8** in $CDCl_3$.

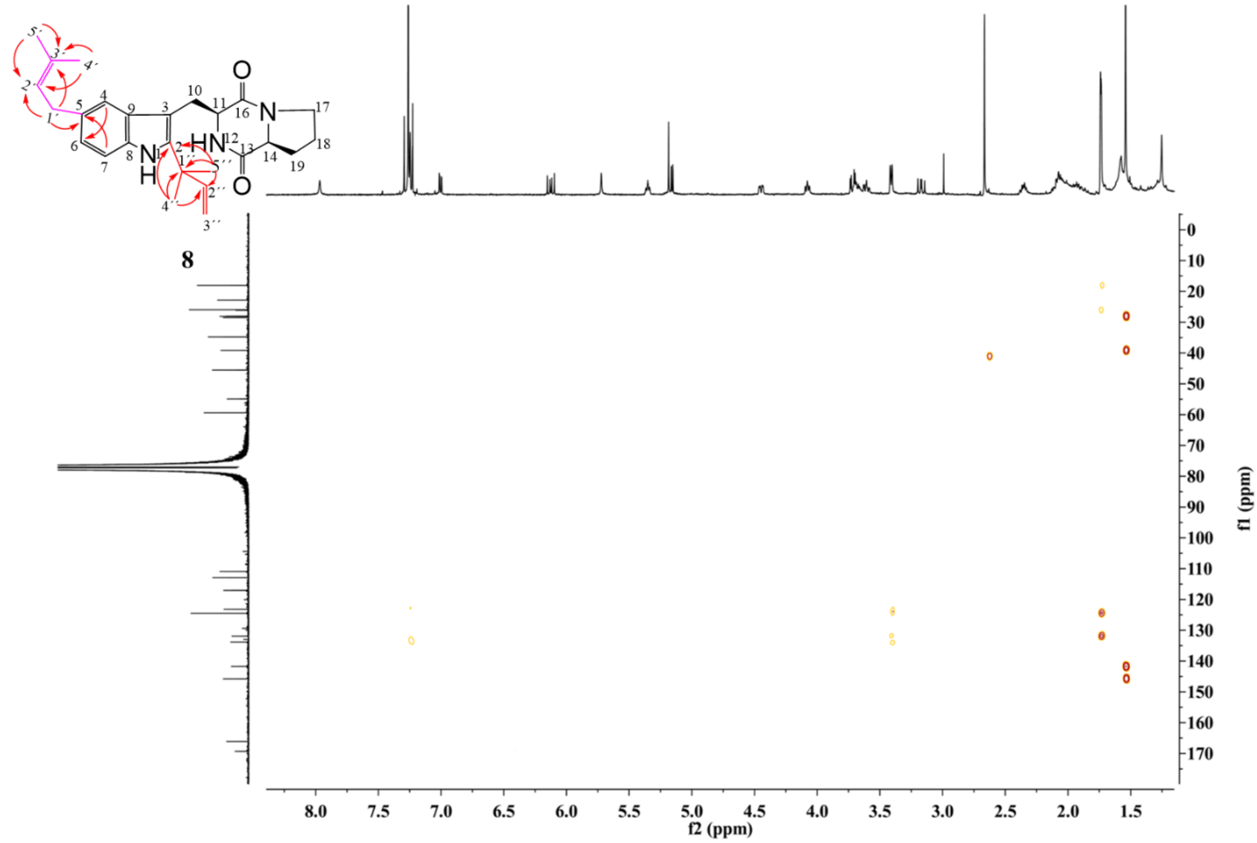

Fig. S19 HMBC spectrum of **8** in $CDCl_3$.

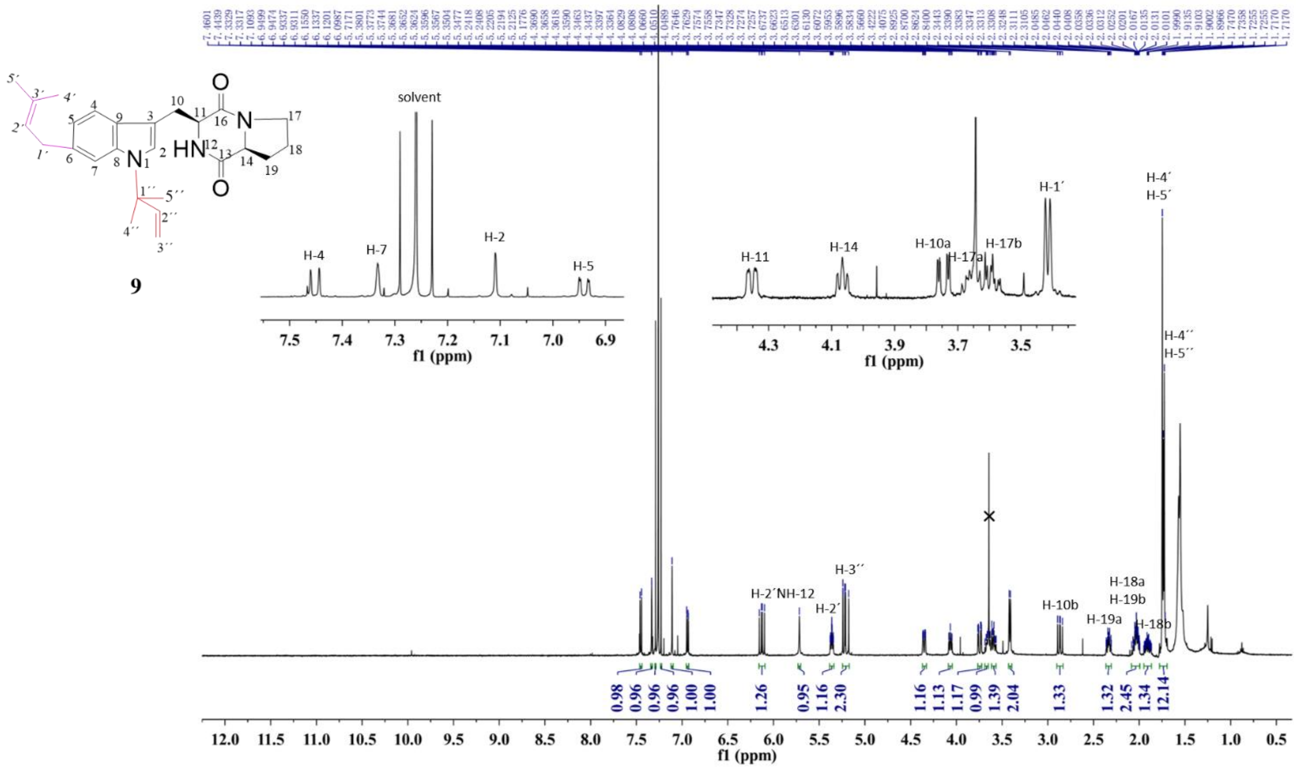

Fig. S20 ^1H NMR spectrum of **9** in $CDCl_3$ (500 MHz).

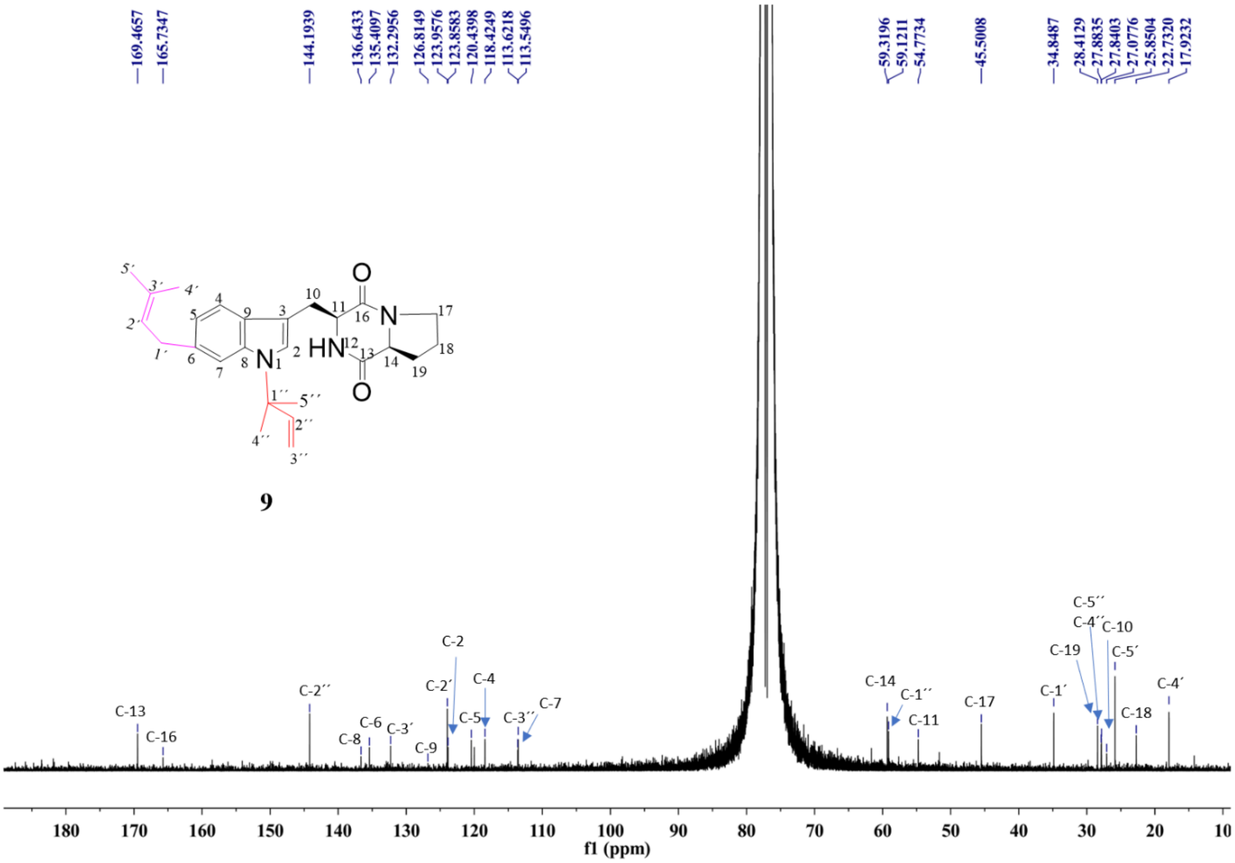

Fig. S21 ^{13}C NMR spectrum of **9** in $CDCl_3$ (125 MHz).

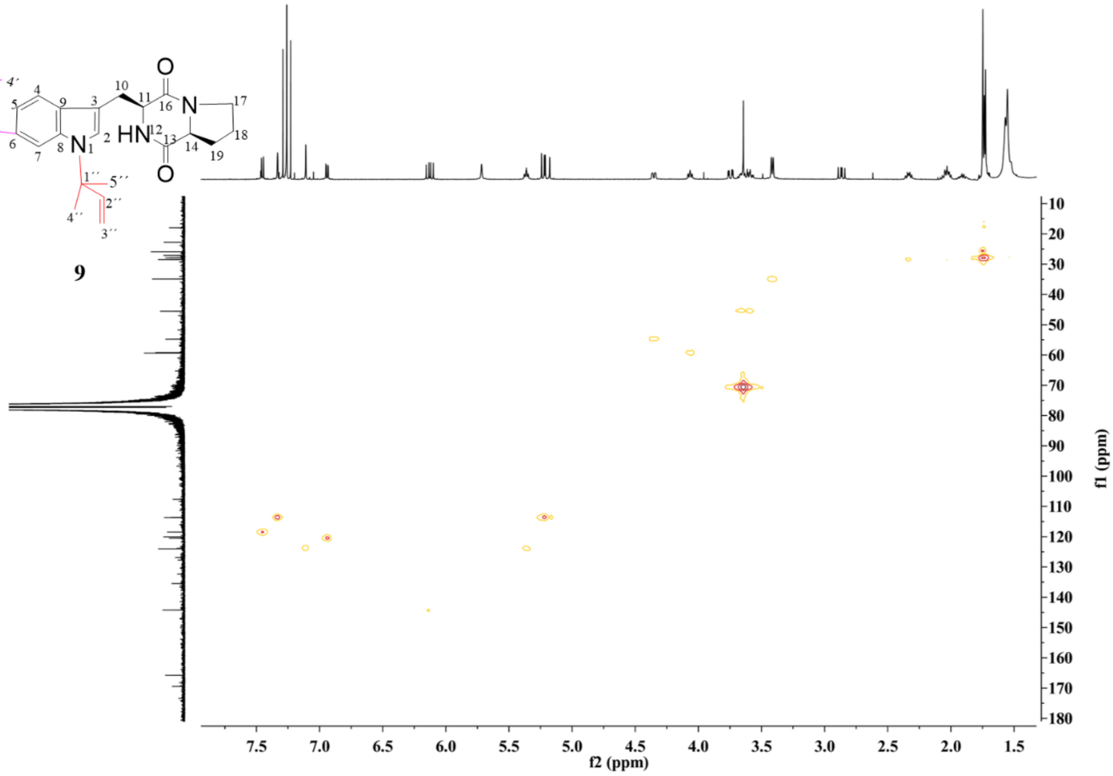

Fig. S22 HSQC spectrum of **9** in $CDCl_3$.

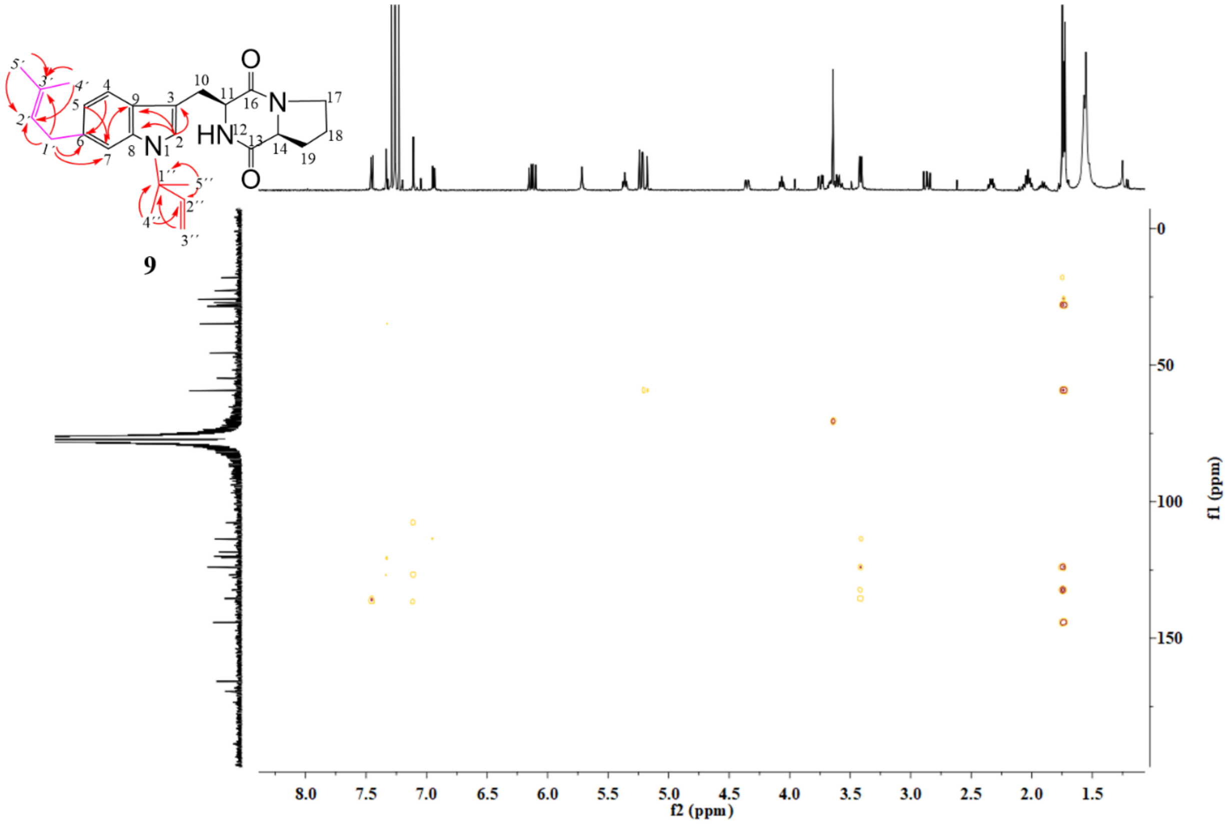

Fig. S23 HMBC spectrum of **9** in $CDCl_3$.

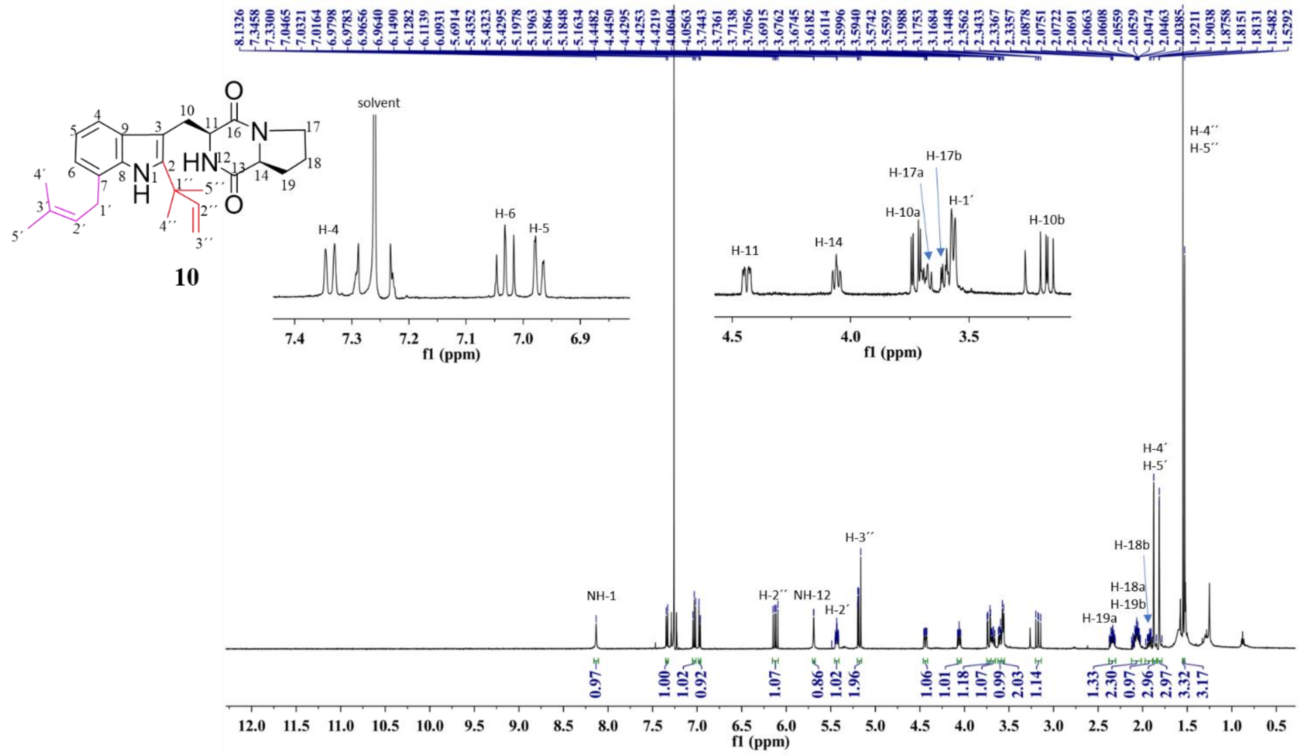

Fig. S24 ^{1}H NMR spectrum of **10** in $CDCl_3$ (500 MHz).

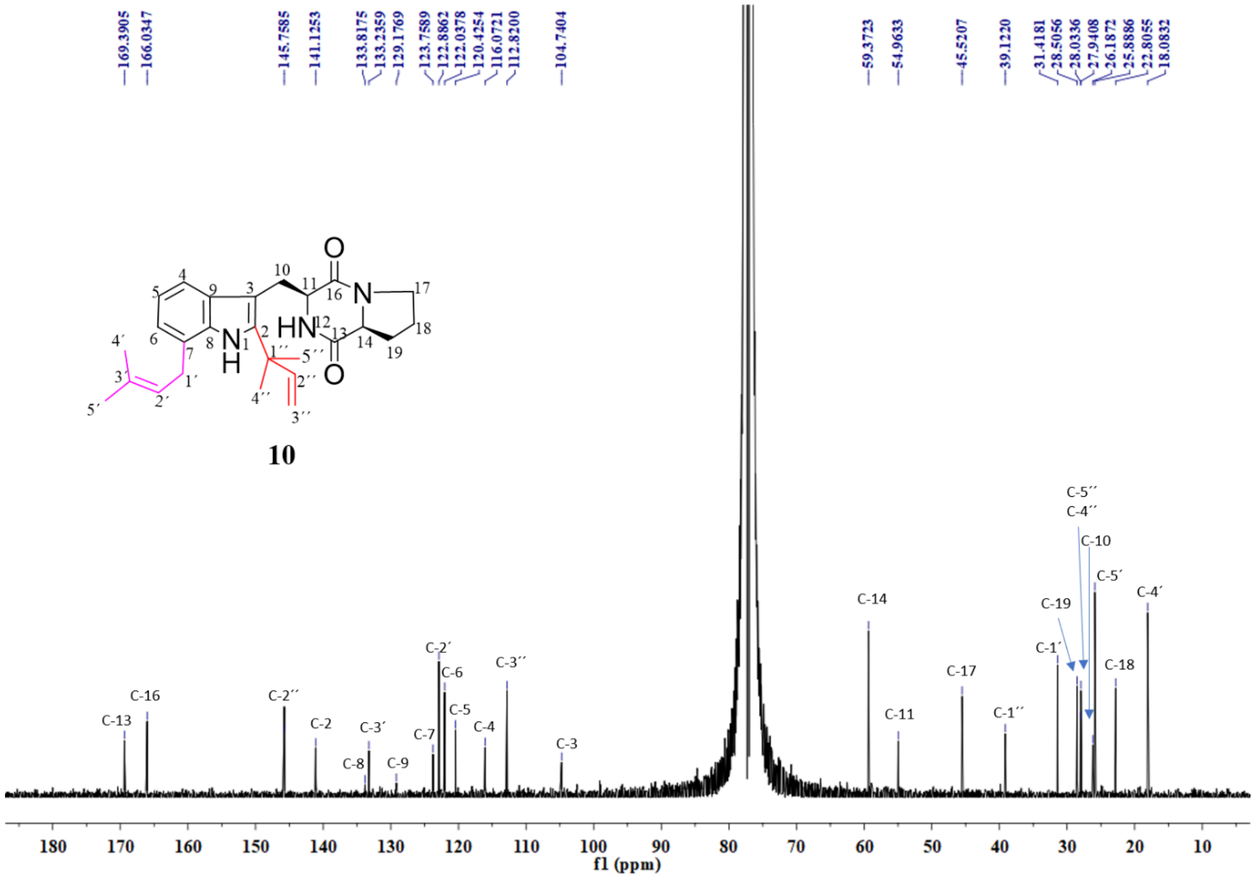

Fig. S25 ^{13}C NMR spectrum of **10** in $CDCl_3$ (125 MHz).

References

Liu R, Zhang H, Wu W, Li H, An Z, Zhou F (2020) C7-Prenylation of tryptophan-containing cyclic dipeptides by 7-dimethylallyl tryptophan synthase significantly increases the anticancer and antimicrobial activities. Molecules 25:3676.

Schkeryantz JM, Woo JCG, Siliphaivanh P, Depew KM, Danishefsky SJ (1999) Total synthesis of gypsetin, deoxybrevianamide E, brevianamide E, and tryprostatin B: novel constructions of 2,3-disubstituted indoles. J Am Chem Soc 121:11964-11975.

Steffan N, Li S-M (2009) Increasing structure diversity of prenylated diketopiperazine derivatives by using a 4-dimethylallyltryptophan synthase. Arch Microbiol 191:461-466.
